# The Revised Medical Care Act is associated with a decrease in hospital death for the total Japanese older adult population regardless of dementia status: An interrupted time series analysis

**DOI:** 10.1371/journal.pone.0264624

**Published:** 2022-03-03

**Authors:** Joost D. Wammes, Miharu Nakanishi, Jenny T. van der Steen, Janet L. MacNeil Vroomen

**Affiliations:** 1 Department of Internal Medicine, Section of Geriatric Medicine, Amsterdam UMC, University of Amsterdam, Amsterdam, The Netherlands; 2 Department of Psychiatric Nursing, Tohoku University Graduate School of Medicine, Miyagi, Japan; 3 Department of Public Health and Primary Care, Leiden University Medical Center, Leiden, The Netherlands; 4 Department of Primary and Community Care, Radboud University Medical Center, Nijmegen, The Netherlands; University of Technology Sydney, AUSTRALIA

## Abstract

**Background:**

In 2006, Japan introduced the Revised Medical Care Act aimed to shift end-of-life care from hospitals to communities. For patients and families, dying in hospital can be highly distressing. Persons with dementia are especially susceptible to negative hospital-related outcomes. This study aims to evaluate whether the Revised Medical Care Act is associated with a decrease in the proportion of hospital deaths for older adults and persons with dementia over a 20-year period covering the reform.

**Methods and findings:**

This is a population-level, repeated cross-sectional study using mortality data from Vital Statistics Japan. Participants were Japanese older adults 65 years or older with and without dementia who died between 1996 and 2016. The policy intervention was the 2006 Revised Medical Care Act that increased community care infrastructure. The primary outcome was location of death in hospital, nursing home, home, or elsewhere. The trend in the proportion of location of death, before and after the reforms was estimated using an interrupted time-series analysis. All analyses were adjusted for sex and seasonality. Of the 19,307,104 older adult decedents, 216,442 had dementia identified on their death certificate. Death in nursing home (1.10, 95% CI 1.10–1.10), home (1.08, 95% CI 1.08–1.08), and elsewhere (1.07, 95% CI 1.07–1.07) increased over time compared to hospital deaths for the total population after reform implementation. Nursing home (1.04, 95% CI 1.03–1.05) and home death (1.11, 95% CI 1.10–1.12) increased after reform implementation for persons with dementia.

**Conclusion:**

This study provides evidence that the 2006 Revised Medical Care Act was associated with decreased older adults dying in hospital regardless of dementia status; however, hospital continues as the primary location of death.

## Introduction

Japan has the largest population of older adults in the world with over 35 million people of 65 years and older [1]. It also has the highest proportion of hospital deaths in the world [2] despite preferences of older Japanese adults to die at home [3]. Dying in a hospital can be highly stressful for patients and families [4] as hospitals rarely provide individualized care required for a positive death experience [5, 6].

Over the last decades, care for older adults in Japan was mainly concentrated in hospitals as they provided large amounts of long-term care. Most community-based long-term care is provided by private healthcare organizations, funded by public insurance schemes [7]. However, long-term care placement was associated with lengthy waiting lists [8]. Moreover, in 2003, 64% of nursing home residents were transferred to hospital for end-of-life care [9].

In 2006, the Japanese government implemented the Revised Medical Care Act, to increase financial sustainability of the health system, and improve care for its aging society. The reform focused on shifting long-term care from in-hospital towards community-based care. Likewise, policies adopted strategies for end-of-life care to take place outside of hospitals, recognizing home and nursing home as preferred location of death [10]. To enable older adults to extend their time at home, the Revised Medical Care Act established end-of-life home care clinics, home hospice services, and expanded in-home social and medical care services under the insurance program. Furthermore, to increase time until death in nursing homes, financial incentives to prevent hospitalizations at the end of life, and additional benefits to pay for end-of-life care in nursing homes were instituted [11, 12].

Sato et al. [13] found that the proportion of hospital death in older adults was steadily decreasing between 2007 to 2014, however, the analysis did not model pre-reform trends, making it difficult to assess if the Revised Medical Care Act was associated with changes in location of death. Furthermore, no studies have yet analyzed the effect of the Revised Medical Care Act on location of death for persons with dementia, who make up a significant amount of the older population [14]. Persons with dementia have complex needs regarding end-of-life care, and hospitalization of this population is associated with increased mortality, functional decline, delirium, and decreased quality of life [15, 16].

This study aims to evaluate the impact of the 2006 Revised Medical Care Act on location of death for the total population older than 65 years with and without dementia using population-level data. We hypothesize that the Revised Medical Care Act would have an initial, small increase in nursing home deaths due to the immediate financial incentives implemented to avoid hospital transfers in the end of life [17], and a larger, gradual decrease in hospital deaths over time because of building the infrastructure for social and health care at community level, enabling greater access to end-of-life care alternatives. Analyzing the effect of policy reforms on location of death is an important indicator of the quality of end-of-life care [18] and provides a better understanding for countries where end-of-life care is increasingly integrated.

## Methods

### Study design

We performed an interrupted time series analysis (ITS) using the guidelines from Bernal et al. [19] to evaluate whether the Revised Medical Care Act implemented April 1, 2006 was associated with changes in location of death for the total Japanese population 65 years and older, and for persons with dementia. We used repeated cross-sectional, open-access, fully anonymized, national-level aggregated data on location of death from Vital Statistics Japan, covering the total Japanese population (S1 File). We used quarterly data on location of death data from January 1, 1996 to March 31, 2006 as the pre-reform period, and from April 1, 2006 to December 31, 2016 as the post-reform period. This study was developed methodical and reporting recommendations for interrupted time series studies by Jandoc et al. [20] (S1 Appendix).

### Participants

Older adults included in this study were 65 years and older at the time of death. Persons with dementia were identified from the death certificates by the codes F00.0, F00.1, F00.2, F00.9, F01.0, F01.1, F01.2, F01.3, F01.8, F01.9, F02.0, F02.1, F02.2, F02.3, F02.4, F02.8, F03, G30.0, G30.1, G30.8, and G30.9 of the International Classification of Diseases, Tenth Revision.

### Variables

The primary outcome was location of death as indicated on the death certificate. Options were death at hospital, nursing home, home, or elsewhere. Death elsewhere mostly referred to death at a rehabilitation center, however, to a lesser extent, unknown location of death. Death in hospice was coded as hospital death because most hospice services in Japan are in-hospital [21].

Explanatory variables included a *Time* variable in cumulative quarters (continuous), a *Reform* dummy variable (The 2006 Revised Medical Care Act) coded as zero pre-reform period and one as post-reform period. Calendar quarters was included as a categorical variable to account for seasonality [19]. Sex was included as a covariate.

### Statistical analysis

Descriptive statistics were used to tabulate unadjusted yearly proportions of location of death for the total population and persons with dementia. Summaries and bivariate comparisons between the outcomes and possible time-varying confounders, and before-and-after reform comparisons were conducted.

Two multinomial logistic regressions were performed for the total population and for persons with dementia to calculate adjusted relative risk ratios (aRRR). The regression analyses were weighted to adjust for population growth over the study period. Year-specific mean predicted probabilities of location of death holding all other variables at their means were used to plot the location of death versus time for the total population and for persons with dementia. Death in hospital was the reference group. Each model included time in cumulative quarters since the start of the study, a reform variable and an interaction term between the reforms and cumulative quarters variable. The cumulative quarters can be interpreted as the quarterly aRRR of dying in a particular location prior to the Revised Medical Care Act. The reform variable can be interpreted as the immediate (step) change following the introduction of the Revised Medical Care Act. The interaction between the cumulative quarters and the reform can be interpreted as the quarterly change in relative risk of dying at a particular location since the introduction the Revised Medical Care Act (slope change). Although interrupted time series are generally unaffected by confounding variables that remain fairly constant over time [19], we adjusted all regression models for seasonality and sex. All analyses were conducted in Stata 16.0. Statistical significance was set at 2-tailed P < 0.05.

## Results

### Descriptive analysis

Between January 1, 1996 and December 31, 2016, a total of 19,307,104 older adults aged 65 years and older were identified from death certificates records from vital statistics Japan. Of these, 50.5% were female. The pre-reform proportions for the total population (N = 8,035,104) based on location of death were hospital 81.8%, home 14.1%, nursing home 2.3%, and elsewhere 1.8%. Post-reform proportions were hospital 80.0%, home 11.6%, nursing home 5.1% death, and elsewhere 3.3%. Table 1 presents yearly proportions of location of death.

**Table 1:** Yearly proportions of location of death (1996–2016) for the total population of Japanese adults over 65 years and for persons with dementia.

	Total population					Persons with dementia				
Years	N	Hospital	Home	Nursing Home	Elsewhere	N	Hospital	Home	Nursing Home	Elsewhere
		(%)	(%)	(%)	(%)		(%)	(%)	(%)	(%)
1996	690,132	78.20	18.17	2.08	1.55	3,098	52.32	35.73	8.91	3.03
1997	710,454	79.10	17.14	2.15	1.62	3,025	54.31	32.89	9.52	3.27
1998	729,851	79.63	16.48	2.15	1.73	3,178	53.37	32.16	10.60	3.87
1999	775,115	80.91	15.25	2.16	1.69	3,686	57.51	27.37	10.80	4.31
2000	761,214	82.06	13.87	2.33	1.74	4,133	55.75	26.37	12.48	5.40
2001	775,297	82.42	13.31	2.44	1.82	4,558	57.83	22.36	13.71	6.10
2002	790,764	82.87	12.96	2.37	1.80	4,676	59.28	20.34	14.50	5.88
2003	822,903	83.33	12.46	2.39	1.82	4,923	60.63	19.11	13.67	6.58
2004	838,215	83.89	11.70	2.54	1.87	5,459	61.71	17.02	14.43	6.83
2005	892,450	84.04	11.47	2.60	1.89	5,826	61.50	15.17	15.98	7.35
2006 ^a^	899,991	83.88	11.28	2.82	2.02	6,960	61.75	15.06	15.79	7.40
2007 ^a^	928,012	83.44	11.37	3.04	2.15	7,973	60.30	13.81	17.27	8.62
2008 ^a^	965,381	82.55	11.71	3.43	2.31	9,309	58.43	13.99	18.42	9.16
2009 ^a^	969,414	82.32	11.36	3.79	2.54	10,214	57.48	12.84	19.95	9.73
2010 ^a^	1,024,572	81.57	11.52	4.10	2.81	12,011	54.92	12.06	21.26	11.76
2011 ^a^	1,072,197	80.09	11.39	4.65	3.87	14,808	54.06	11.35	22.82	11.78
2012 ^a^	1,093,233	79.54	11.78	5.32	3.36	17,714	52.05	11.67	23.80	12.47
2013 ^a^	1,113,255	78.56	11.81	6.00	3.63	20,494	51.47	11.48	25.33	11.72
2014 ^a^	1,126,449	77.93	11.75	6.50	3.82	22,573	50.40	10.88	26.32	12.40
2015 ^a^	1,152,817	77.16	11.70	7.07	4.07	24,637	49.51	10.52	27.14	12.83
2016 ^a^	1,175,388	76.26	11.97	7.65	4.13	27,187	48.08	10.57	28.82	12.53
Total	19,307,104					216,442				

^a^ Indicates post-reform period

From the total population, 216,442 (1.1%) were identified with dementia as the main cause of death; 67.0% were female. The pre-reform location of death proportions (N = 44,430) were hospital 58.2%, home 23.1%, nursing home 13.0%, and elsewhere 5.6%. Post reform (N = 172,013) proportions were hospital 52.5%, home 11.6%, nursing home 24.3%, and elsewhere 11.7%.

### Multinomial logistic regressions

#### Total population

**Fig 1:**
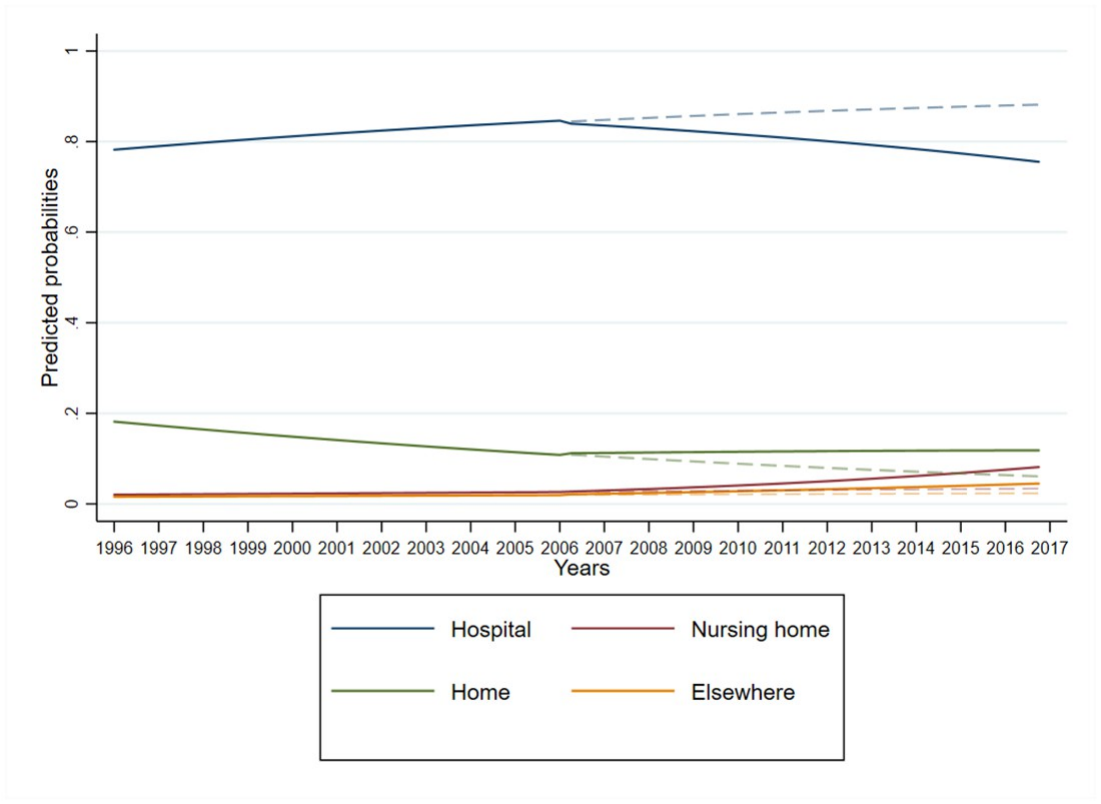
Quarterly predicted probabilities of location of death plotted over time for the total Japanese population over 65 years old.

Based on the estimates from the interrupted time series analysis (Fig 1, Table 2), prior to the introduction of the Revised Medical Care Act, the proportions of people dying in nursing homes (aRRR 1.02, 95% CI 1.02–1.02) and elsewhere (aRRR 1.01, 95% CI 1.01–1.01) were significantly increasing compared to hospital death, whereas home death was significantly decreasing (aRRR 0.94, 95% CI 0.94–0.94). After the introduction of the Revised Medical Care Act, there was a small, negative step change in the proportion of people dying in nursing homes (aRRR 0.37, 95% CI 0.37–0.38), at home (aRRR 0.49, 95% CI 0.48–0.49), and elsewhere (aRRR 0.53, 95% CI 0.52–0.54) compared to hospital deaths. However, over time, we found that the implementation of the Revised Medical Care Act showed a modest significant acceleration (i.e., increased slope) in nursing homes deaths (aRRR 1.10, 95% CI 1.10–1.10), home (aRRR 1.08, 95% CI 1.08–1.08), and elsewhere (aRRR 1.07, 95% CI 1.07–1.07). Females were more likely than males to die in nursing home (aRRR 3.09, 95% CI 3.08–3.11), home (aRRR 1.16, 95% CI 1.16–1.16) or elsewhere (aRRR 1.50, 95% CI 1.49–1.50) than in a hospital.

**Table 2:** Interrupted time series analysis of year quarters, adjusted relative risk ratios of location of death for the total Japanese population 65 years and older, and for persons with dementia.

	Total population			Dementia		
	N = 19,307,104			N = 216,442		
	Nursing home	Home	Elsewhere	Nursing home	Home	Elsewhere
	aRRR	aRRR	aRRR	aRRR	aRRR	aRRR
	(95% CI)	(95% CI)	(95% CI)	(95% CI)	(95% CI)	(95% CI)
	p-value	p-value	p-value	p-value	p-value	p-value
pre- intervention trend	1.02	0.94	1.01	1.04	0.89	1.07
	(1.02–1.02)	(0.94–0.94)	(1.01–1.01)	(1.03–1.05)	(0.88–0.89)	(1.05–1.09)
	< 0.001	< 0.001	< 0.001	< 0.001	< 0.001	< 0.001
Step change after the Revised Medical Care Act	0.37	0.49	0.53	0.66	0.34	1.22
	(0.37–0.38)	(0.48–0.49)	(0.52–0.54)	(0.60–0.73)	(0.30–0.37)	(1.05–1.40)
	< 0.001	< 0.001	< 0.001	< 0.001	< 0.001	0.007
Slope change after the Revised Medical Care Act	1.10	1.08	1.07	1.04	1.11	0.99
	(1.10–1.10)	(1.08–1.08)	(1.07–1.07)	(1.03–1.05)	(1.10–1.12)	(0.98–1.01)
	< 0.001	< 0.001	< 0.001	< 0.001	< 0.001	0.529
Females	3.09	1.16	1.50	2.87	1.54	2.12
	(3.08–3.11)	(1.16–1.16)	(1.49–1.50)	(2.79–2.94)	(1.49–1.58)	(2.05–2.19)
	< 0.001	< 0.001	< 0.001	< 0.001	< 0.001	< 0.001

Data are year quarters, adjusted Relative Risk Ratios (aRRR) from weighted, multinomial logistic regressions with hospital death set as reference category.

#### Persons with dementia

**Fig 2:**
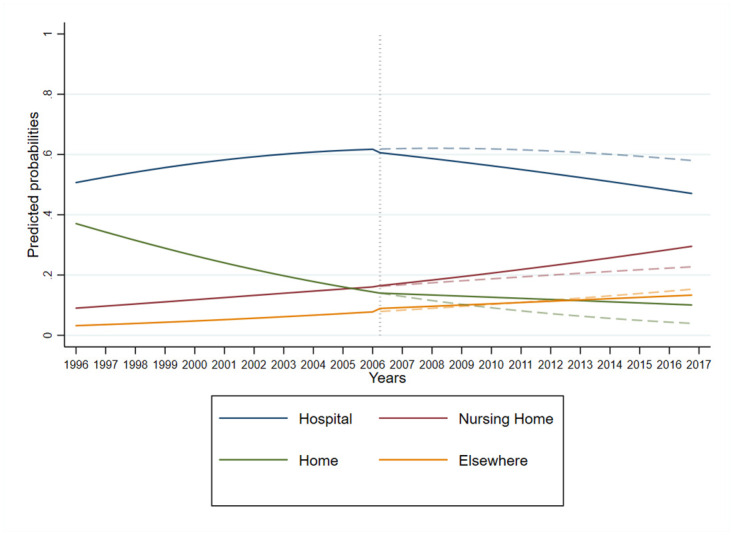
Quarterly predicted probabilities of location of death plotted over time for persons with dementia.

Before the introduction of the Revised Medical Care Act, estimates from the interrupted time-series indicated for persons with dementia, a significant increase in nursing home death (aRRR 1.04, 95% CI 1.03–1.05) and death elsewhere (aRRR 1.07, 95% CI 1.05–1.09) compared to hospital death, while home death decreased (aRRR 0.89, 95% CI 0.88–0.89) (Fig 2, Table 2). Immediately after the implementation of the Revised Medical Care Act, there was a small yet significant negative step change in the proportion of people with dementia dying in a nursing home (aRRR 0.66, 95% CI 0.60–0.73) and home (aRRR 0.34, 95% CI 0.30–0.37), while there was a significant positive step change in death elsewhere (aRRR 1.22, 95% CI 1.05–1.40) versus hospital death. This was followed by an acceleration in slope for death in nursing home (aRRR 1.04, 95% CI 1.03–1.05) and at home (aRRR 1.11, 95% CI 1.10–1.12). Females were more likely than males to die in a nursing home (aRRR 2.87, 95% CI 2.79–2.94), home (aRRR 1.54, 95% CI 1.49–1.58) or elsewhere (aRRR 2.12, 95% CI 2.05–2.19) than in a hospital.

## Discussion

With the immediate introduction of the Revised Medical Care Act, in contrast to our hypothesis, there was a slight increase in risk in hospital death for the Japanese older adult population despite financial incentives to avoid hospital transfers in the end of life. Consistent with our hypothesis, we found that over time, the Revised Medical Care act was associated with a significant shift in the proportions of death in hospital towards nursing home, home and elsewhere. For persons with dementia, there was also a slight immediate increased risk of hospital death at the time of the Revised Medical Care Act introduction. However, over time nursing home deaths significantly increased while hospital deaths gradually decreased. Although there was an acceleration in the slope of home deaths for the total population and for persons with dementia, home deaths did not return to pre-reform levels. Hospital remained the primary location of death for the total older adult population and for persons with dementia.

The substitution of hospital deaths towards nursing home, home, and elsewhere may be related to specific components of the Revised Medical Care Act. First, since the reform implementation payment of hospital beds was restricted to patients with high medical care needs, while those with lower care needs were transferred to nursing homes or home. Subsequently, the number of long-term care beds in hospitals were reduced from around 383,000 in October 2005 to 316,000 in 2019 [22]. Second, there was an expansion of nursing home facility beds over time. Which has steadily increased the amount of older individuals in nursing homes to 179 per 1000 in 2012 [23]. Moreover, the Revised Medical Care Act has implemented financial incentives to stimulate end-of-life care in nursing homes. For residents that were physically in the nursing home, there was a per diem bonus of 9 dollar for 30 to 4 days before death, and an extra 142-dollar per diem from 3 days to the date of death, both on top of the 89-dollar standard daily rate [24]. Third, aging in place was stimulated by the introduction of Community General Support Centers at community level that aimed to prevent admission to long-term care facilities, through providing comprehensive support for home-dwelling older adults such as home health care, housing improvements, and housing provision [25]. Fourth, Home Care Supporting Clinics were introduced, which had the purpose to play a central role in end-of-life care at home [26]. These clinics should be able to provide a 24 hour a day home hospice function in cooperation with hospitals, and assign care managers to older adults with a palliative status. In 2011, over 11,775 Home Care Supporting Clinics were set up, of which 90% operated following end-of-life care procedures. Last, for persons with dementia specifically, small group homes, day care services, and night care were established under the Revised Medical Care Act [27].

The Revised Medical Care Act policy reform is comparable to the 2012 Norwegian Coordination Reform that aimed at extending time at home by building infrastructure to enable in-home care through community services. MacNeil—Vroomen et al. [28] found for the total older adult population and for persons with dementia that the Norwegian reform was associated with a decrease in proportions of hospital death and an increase in nursing home deaths, however, the number of persons dying at home did not change [28]. The 2015 long-term care reforms from the Netherlands, that also aimed at keeping persons in the community for as long as possible, implemented austerity measures such as closing long-term care facilities and cutting the home care budget [29]. Although the Netherlands has one of the lowest proportions of hospital deaths in the world, the reforms were associated with an increase in hospital and home deaths for the total population and for persons with dementia [29].

While this study found that the proportion of nursing home deaths has approximately doubled for the total Japanese population and for persons with dementia since the Revised Medical Care Act, a far larger proportion of persons with dementia died in a nursing home compared to the total population. The difference in proportions might be explained by persons with dementia entering nursing homes earlier than older adults without dementia [30]. In Japan many older adults are on waiting lists for nursing home placement (523,584 older adults in 2014) [8], and nursing homes have limited capacity for providing care. Japanese older adults with highly complex care needs such dementia are prioritized to nursing homes [30].

The proportion of nursing home death in Japan remain relatively low compared to Norway (total population 57.3%, dementia 90.5% [28]) and the Netherlands (total population 29.5%, dementia 89.9% [29]). Gao [31] proposes a framework to guide the evaluation of the role of the health care systems in location of death, and stresses the importance of end-of-life policies. Yet, recent evidence shows that many national health policies in aging societies around the world lack specific reference to end-of-life care [32]. Consequently, the low proportions of nursing home death in Japan might be attributed to the lack of end-of-life policies. Ikegami et al. [17] found, from a sample of Japanese nursing homes, that 35.3% were not registered as a designated end-of-life care facility. Moreover, only 29.9% of the nursing homes had a basic policy to provide end-of-life care, while the policy of 35.6% was a hospital transfer, and 34.5% had no explicit end-of-life policy. Nursing homes that were registered as end-of-life care facility and nursing homes with a basic end-of-life care policy had substantially more deaths within the facility [17]. In contrast, in Norway and the Netherlands end-of-life policies such as advance care planning and do-not-hospitalize are generally well established in nursing homes, resulting in few hospitalizations at the end of life [33, 34].

Despite home death is the most preferred location of death in Japan [3], we found that after the Revised Medical Care Act implementation, the proportion of home deaths for the total population remained relatively the same over time and for persons with dementia was still in a slight downward trend. These results could be a consequence of Japanese persons regard home death as a burden to their families, and many family members prefer a transfer in the end of life to hospital as this is very common [35]. Moreover, evidence shows middle aged Japanese prefer hospital care over family and professional home care if acquired dementia later in life [36]. Yet, the main obstacle may be the lack of home services that support end-of-life care available to older adults and their family [11, 37].

This study found that proportions of death elsewhere increased after the enactment of the Revised Medical Care Act for the total population and persons with dementia. Death elsewhere typically refers to death at a rehabilitation center. An increase of death elsewhere might be a result of older adults admitted to rehabilitation centers as a form of intermediate care because of scarce nursing home placements. Moreover, many older adults are often never discharged from rehabilitation center to home or nursing home [38].

Strengths of this study include being the first study to evaluate effect of the Revised Medical Care Act on location of death. Further, we used repeated, annual cross-sectional data from the entire Japanese older adult population spanning ten years of post- and pre-reform data. Location of death data reliability was assumed because it is legally mandated by Japanese law to be recorded by a medical doctor who has attended the patient. Limitations include death certificates underestimating the number of deaths with dementia. Death certificates are known for underreporting dementia occurrence [39]. The Japanese older adult population prevalence of dementia and related diseases is estimated at 11.3% [40], compared to 1.1% reported in this study. Nevertheless, this was the only identification method available on Japanese population level. Furthermore, there was no data on severity of cognitive impairment for persons with dementia.

This study shows that the 2006 Revised Medical Care Act was associated with a decrease in older adults and persons with dementia dying in hospital. In the years preceding 2006, health for older adults mainly focused on medical care aspects, and hospitals provided a substantial amount of long-term care. In contrast, the Revised Medical Care Act was characterized by integrated measures across health and social care for older adults at community level. This approach appears as a key consideration to improving aging in place because it was successful in modestly decreasing hospital death. However, building sufficient capacity remains an important challenge for a society that is aging rapidly. For this reason, the Revised Medical Care Act might have been suboptimal in improving aging in place, as hospital death was still the primary location of death. The lack of availability of social care might have hampered extending the time in the community until death for older adults [11]. Because care needs of older adults increase over time, health professionals, family members or older adults might choose hospitalization in the end of life when home care, advocacy services and family support is not well integrated or available at community level. Another important issue may have been insufficient availability of nursing home beds. Moreover, once admitted to a nursing home, due to the absence of well-established end-of-life strategies such as advance care planning, older adults were likely to experience hospital transfers at the end of life [17].

Dementia is characterized by gradual loss of decisional capacity that may lead to crisis for them and their families at some points, making their end-of-life care needs distinct from other populations. For persons with dementia, the long trajectory towards the end of life encompasses complex care needs that require continuity and coordination of care across health and social care services. Therefore, we recommend policy that enables proactive end-of-life care planning that is integrated into health and social care reforms [41].

## Supporting information

S1 File (PDF)

S1 Appendix (DOCX)
