# Supplementary material for: The Revised Medical Care Act is associated with a decrease in hospital death for the total Japanese older adult population regardless of dementia status: An interrupted time series analysis
Source: PLoS One. 2022 Mar 3;17(3):e0264624. doi: 10.1371/journal.pone.0264624 (PMC8893620; doi:10.1371/journal.pone.0264624)
Supplement: S1 File — (PDF) [file pone.0264624.s001.pdf]

id,y,elapsed_quarters,years_trend,national_plan,quarters,cog_status,pop,man,years
1,LTCF,1,.25,0,Jan-Mar,no dementia,1226,male,1996
1,home,1,.25,0,Jan-Mar,no dementia,16927,male,1996
1,hospital,1,.25,0,Jan-Mar,no dementia,75677,male,1996
1,elsehwere,1,.25,0,Jan-Mar,no dementia,1451,male,1996
2,LTCF,2,.5,0,Apr-June,no dementia,990,male,1996
2,home,2,.5,0,Apr-June,no dementia,12204,male,1996
2,hospital,2,.5,0,Apr-June,no dementia,68371,male,1996
2,elsehwere,2,.5,0,Apr-June,no dementia,1298,male,1996
3,LTCF,3,.75,0,July-Sept,no dementia,836,male,1996
3,home,3,.75,0,July-Sept,no dementia,11083,male,1996
3,hospital,3,.75,0,July-Sept,no dementia,65176,male,1996
3,elsehwere,3,.75,0,July-Sept,no dementia,1207,male,1996
4,LTCF,4,1,0,Oct-Dec,no dementia,1076,male,1996
4,home,4,1,0,Oct-Dec,no dementia,14914,male,1996
4,hospital,4,1,0,Oct-Dec,no dementia,74282,male,1996
4,elsehwere,4,1,0,Oct-Dec,no dementia,1577,male,1996
5,LTCF,5,1.25,0,Jan-Mar,no dementia,1399,male,1997
5,home,5,1.25,0,Jan-Mar,no dementia,17371,male,1997
5,hospital,5,1.25,0,Jan-Mar,no dementia,83894,male,1997
5,elsehwere,5,1.25,0,Jan-Mar,no dementia,1588,male,1997
6,LTCF,6,1.5,0,Apr-June,no dementia,986,male,1997
6,home,6,1.5,0,Apr-June,no dementia,11966,male,1997
6,hospital,6,1.5,0,Apr-June,no dementia,70031,male,1997
6,elsehwere,6,1.5,0,Apr-June,no dementia,1415,male,1997
7,LTCF,7,1.75,0,July-Sept,no dementia,836,male,1997
7,home,7,1.75,0,July-Sept,no dementia,10681,male,1997
7,hospital,7,1.75,0,July-Sept,no dementia,66606,male,1997
7,elsehwere,7,1.75,0,July-Sept,no dementia,1305,male,1997
8,LTCF,8,2,0,Oct-Dec,no dementia,1206,male,1997
8,home,8,2,0,Oct-Dec,no dementia,14154,male,1997
8,hospital,8,2,0,Oct-Dec,no dementia,74986,male,1997
8,elsehwere,8,2,0,Oct-Dec,no dementia,1685,male,1997
9,LTCF,9,2.25,0,Jan-Mar,no dementia,1392,male,1998
9,home,9,2.25,0,Jan-Mar,no dementia,16906,male,1998
9,hospital,9,2.25,0,Jan-Mar,no dementia,84836,male,1998
9,elsehwere,9,2.25,0,Jan-Mar,no dementia,1811,male,1998
10,LTCF,10,2.5,0,Apr-June,no dementia,927,male,1998
10,home,10,2.5,0,Apr-June,no dementia,11640,male,1998
10,hospital,10,2.5,0,Apr-June,no dementia,71461,male,1998
10,elsehwere,10,2.5,0,Apr-June,no dementia,1573,male,1998
11,LTCF,11,2.75,0,July-Sept,no dementia,920,male,1998
11,home,11,2.75,0,July-Sept,no dementia,11269,male,1998
11,hospital,11,2.75,0,July-Sept,no dementia,69860,male,1998
11,elsehwere,11,2.75,0,July-Sept,no dementia,1490,male,1998
12,LTCF,12,3,0,Oct-Dec,no dementia,1142,male,1998
12,home,12,3,0,Oct-Dec,no dementia,14177,male,1998
12,hospital,12,3,0,Oct-Dec,no dementia,79587,male,1998
12,elsehwere,12,3,0,Oct-Dec,no dementia,1731,male,1998
13,LTCF,13,3.25,0,Jan-Mar,no dementia,1496,male,1999

13,home,13,3.25,0,Jan-Mar,no dementia,18083,male,1999
13,hospital,13,3.25,0,Jan-Mar,no dementia,98494,male,1999
13,elsehwere,13,3.25,0,Jan-Mar,no dementia,1904,male,1999
14,LTCF,14,3.5,0,Apr-June,no dementia,1000,male,1999
14,home,14,3.5,0,Apr-June,no dementia,11320,male,1999
14,hospital,14,3.5,0,Apr-June,no dementia,76190,male,1999
14,elsehwere,14,3.5,0,Apr-June,no dementia,1542,male,1999
15,LTCF,15,3.75,0,July-Sept,no dementia,908,male,1999
15,home,15,3.75,0,July-Sept,no dementia,10489,male,1999
15,hospital,15,3.75,0,July-Sept,no dementia,71929,male,1999
15,elsehwere,15,3.75,0,July-Sept,no dementia,1491,male,1999
16,LTCF,16,4,0,Oct-Dec,no dementia,1209,male,1999
16,home,16,4,0,Oct-Dec,no dementia,13386,male,1999
16,hospital,16,4,0,Oct-Dec,no dementia,81601,male,1999
16,elsehwere,16,4,0,Oct-Dec,no dementia,1721,male,1999
17,LTCF,17,4.25,0,Jan-Mar,no dementia,1376,male,2000
17,home,17,4.25,0,Jan-Mar,no dementia,15905,male,2000
17,hospital,17,4.25,0,Jan-Mar,no dementia,94669,male,2000
17,elsehwere,17,4.25,0,Jan-Mar,no dementia,1738,male,2000
18,LTCF,18,4.5,0,Apr-June,no dementia,1087,male,2000
18,home,18,4.5,0,Apr-June,no dementia,10168,male,2000
18,hospital,18,4.5,0,Apr-June,no dementia,77418,male,2000
18,elsehwere,18,4.5,0,Apr-June,no dementia,1655,male,2000
19,LTCF,19,4.75,0,July-Sept,no dementia,933,male,2000
19,home,19,4.75,0,July-Sept,no dementia,9585,male,2000
19,hospital,19,4.75,0,July-Sept,no dementia,73444,male,2000
19,elsehwere,19,4.75,0,July-Sept,no dementia,1476,male,2000
20,LTCF,20,5,0,Oct-Dec,no dementia,1217,male,2000
20,home,20,5,0,Oct-Dec,no dementia,12431,male,2000
20,hospital,20,5,0,Oct-Dec,no dementia,82840,male,2000
20,elsehwere,20,5,0,Oct-Dec,no dementia,1793,male,2000
21,LTCF,21,5.25,0,Jan-Mar,no dementia,1398,male,2001
21,home,21,5.25,0,Jan-Mar,no dementia,14249,male,2001
21,hospital,21,5.25,0,Jan-Mar,no dementia,89185,male,2001
21,elsehwere,21,5.25,0,Jan-Mar,no dementia,1863,male,2001
22,LTCF,22,5.5,0,Apr-June,no dementia,1137,male,2001
22,home,22,5.5,0,Apr-June,no dementia,10534,male,2001
22,hospital,22,5.5,0,Apr-June,no dementia,81081,male,2001
22,elsehwere,22,5.5,0,Apr-June,no dementia,1768,male,2001
23,LTCF,23,5.75,0,July-Sept,no dementia,1028,male,2001
23,home,23,5.75,0,July-Sept,no dementia,9874,male,2001
23,hospital,23,5.75,0,July-Sept,no dementia,77093,male,2001
23,elsehwere,23,5.75,0,July-Sept,no dementia,1496,male,2001
24,LTCF,24,6,0,Oct-Dec,no dementia,1317,male,2001
24,home,24,6,0,Oct-Dec,no dementia,12898,male,2001
24,hospital,24,6,0,Oct-Dec,no dementia,87287,male,2001
24,elsehwere,24,6,0,Oct-Dec,no dementia,1891,male,2001
25,LTCF,25,6.25,0,Jan-Mar,no dementia,1337,male,2002
25,home,25,6.25,0,Jan-Mar,no dementia,14034,male,2002
25,hospital,25,6.25,0,Jan-Mar,no dementia,91703,male,2002

25,elsehwere,25,6.25,0,Jan-Mar,no dementia,1783,male,2002
26,LTCF,26,6.5,0,Apr-June,no dementia,1063,male,2002
26,home,26,6.5,0,Apr-June,no dementia,10350,male,2002
26,hospital,26,6.5,0,Apr-June,no dementia,81489,male,2002
26,elsehwere,26,6.5,0,Apr-June,no dementia,1659,male,2002
27,LTCF,27,6.75,0,July-Sept,no dementia,985,male,2002
27,home,27,6.75,0,July-Sept,no dementia,10078,male,2002
27,hospital,27,6.75,0,July-Sept,no dementia,79172,male,2002
27,elsehwere,27,6.75,0,July-Sept,no dementia,1625,male,2002
28,LTCF,28,7,0,Oct-Dec,no dementia,1245,male,2002
28,home,28,7,0,Oct-Dec,no dementia,13219,male,2002
28,hospital,28,7,0,Oct-Dec,no dementia,91256,male,2002
28,elsehwere,28,7,0,Oct-Dec,no dementia,1899,male,2002
29,LTCF,29,7.25,0,Jan-Mar,no dementia,1420,male,2003
29,home,29,7.25,0,Jan-Mar,no dementia,15364,male,2003
29,hospital,29,7.25,0,Jan-Mar,no dementia,100307,male,2003
29,elsehwere,29,7.25,0,Jan-Mar,no dementia,2015,male,2003
30,LTCF,30,7.5,0,Apr-June,no dementia,1148,male,2003
30,home,30,7.5,0,Apr-June,no dementia,10658,male,2003
30,hospital,30,7.5,0,Apr-June,no dementia,85084,male,2003
30,elsehwere,30,7.5,0,Apr-June,no dementia,1716,male,2003
31,LTCF,31,7.75,0,July-Sept,no dementia,1028,male,2003
31,home,31,7.75,0,July-Sept,no dementia,9969,male,2003
31,hospital,31,7.75,0,July-Sept,no dementia,81077,male,2003
31,elsehwere,31,7.75,0,July-Sept,no dementia,1572,male,2003
32,LTCF,32,8,0,Oct-Dec,no dementia,1208,male,2003
32,home,32,8,0,Oct-Dec,no dementia,12203,male,2003
32,hospital,32,8,0,Oct-Dec,no dementia,91311,male,2003
32,elsehwere,32,8,0,Oct-Dec,no dementia,1911,male,2003
33,LTCF,33,8.25,0,Jan-Mar,no dementia,1439,male,2004
33,home,33,8.25,0,Jan-Mar,no dementia,14385,male,2004
33,hospital,33,8.25,0,Jan-Mar,no dementia,100123,male,2004
33,elsehwere,33,8.25,0,Jan-Mar,no dementia,2014,male,2004
34,LTCF,34,8.5,0,Apr-June,no dementia,1215,male,2004
34,home,34,8.5,0,Apr-June,no dementia,10203,male,2004
34,hospital,34,8.5,0,Apr-June,no dementia,86245,male,2004
34,elsehwere,34,8.5,0,Apr-June,no dementia,1707,male,2004
35,LTCF,35,8.75,0,July-Sept,no dementia,1078,male,2004
35,home,35,8.75,0,July-Sept,no dementia,9729,male,2004
35,hospital,35,8.75,0,July-Sept,no dementia,83786,male,2004
35,elsehwere,35,8.75,0,July-Sept,no dementia,1731,male,2004
36,LTCF,36,9,0,Oct-Dec,no dementia,1392,male,2004
36,home,36,9,0,Oct-Dec,no dementia,12398,male,2004
36,hospital,36,9,0,Oct-Dec,no dementia,96064,male,2004
36,elsehwere,36,9,0,Oct-Dec,no dementia,1977,male,2004
37,LTCF,37,9.25,0,Jan-Mar,no dementia,1667,male,2005
37,home,37,9.25,0,Jan-Mar,no dementia,15732,male,2005
37,hospital,37,9.25,0,Jan-Mar,no dementia,108737,male,2005
37,elsehwere,37,9.25,0,Jan-Mar,no dementia,2168,male,2005
38,LTCF,38,9.5,0,Apr-June,no dementia,1363,male,2005

38,home,38,9.5,0,Apr-June,no dementia,10323,male,2005
38,hospital,38,9.5,0,Apr-June,no dementia,92430,male,2005
38,elsehwere,38,9.5,0,Apr-June,no dementia,1871,male,2005
39,LTCF,39,9.75,0,July-Sept,no dementia,1133,male,2005
39,home,39,9.75,0,July-Sept,no dementia,9445,male,2005
39,hospital,39,9.75,0,July-Sept,no dementia,87695,male,2005
39,elsehwere,39,9.75,0,July-Sept,no dementia,1726,male,2005
40,LTCF,40,10,0,Oct-Dec,no dementia,1505,male,2005
40,home,40,10,0,Oct-Dec,no dementia,13424,male,2005
40,hospital,40,10,0,Oct-Dec,no dementia,100795,male,2005
40,elsehwere,40,10,0,Oct-Dec,no dementia,1994,male,2005
41,LTCF,41,10.25,0,Jan-Mar,no dementia,1627,male,2006
41,home,41,10.25,0,Jan-Mar,no dementia,14680,male,2006
41,hospital,41,10.25,0,Jan-Mar,no dementia,105888,male,2006
41,elsehwere,41,10.25,0,Jan-Mar,no dementia,2156,male,2006
42,LTCF,42,10.5,1,Apr-June,no dementia,1533,male,2006
42,home,42,10.5,1,Apr-June,no dementia,10738,male,2006
42,hospital,42,10.5,1,Apr-June,no dementia,93954,male,2006
42,elsehwere,42,10.5,1,Apr-June,no dementia,1923,male,2006
43,LTCF,43,10.75,1,July-Sept,no dementia,1269,male,2006
43,home,43,10.75,1,July-Sept,no dementia,10189,male,2006
43,hospital,43,10.75,1,July-Sept,no dementia,89510,male,2006
43,elsehwere,43,10.75,1,July-Sept,no dementia,1796,male,2006
44,LTCF,44,11,1,Oct-Dec,no dementia,1611,male,2006
44,home,44,11,1,Oct-Dec,no dementia,12991,male,2006
44,hospital,44,11,1,Oct-Dec,no dementia,100742,male,2006
44,elsehwere,44,11,1,Oct-Dec,no dementia,2242,male,2006
45,LTCF,45,11.25,1,Jan-Mar,no dementia,1768,male,2007
45,home,45,11.25,1,Jan-Mar,no dementia,14823,male,2007
45,hospital,45,11.25,1,Jan-Mar,no dementia,106967,male,2007
45,elsehwere,45,11.25,1,Jan-Mar,no dementia,2282,male,2007
46,LTCF,46,11.5,1,Apr-June,no dementia,1595,male,2007
46,home,46,11.5,1,Apr-June,no dementia,11222,male,2007
46,hospital,46,11.5,1,Apr-June,no dementia,95628,male,2007
46,elsehwere,46,11.5,1,Apr-June,no dementia,2084,male,2007
47,LTCF,47,11.75,1,July-Sept,no dementia,1493,male,2007
47,home,47,11.75,1,July-Sept,no dementia,11166,male,2007
47,hospital,47,11.75,1,July-Sept,no dementia,92437,male,2007
47,elsehwere,47,11.75,1,July-Sept,no dementia,2020,male,2007
48,LTCF,48,12,1,Oct-Dec,no dementia,1810,male,2007
48,home,48,12,1,Oct-Dec,no dementia,14082,male,2007
48,hospital,48,12,1,Oct-Dec,no dementia,105241,male,2007
48,elsehwere,48,12,1,Oct-Dec,no dementia,2337,male,2007
49,LTCF,49,12.25,1,Jan-Mar,no dementia,2075,male,2008
49,home,49,12.25,1,Jan-Mar,no dementia,16738,male,2008
49,hospital,49,12.25,1,Jan-Mar,no dementia,112632,male,2008
49,elsehwere,49,12.25,1,Jan-Mar,no dementia,2530,male,2008
50,LTCF,50,12.5,1,Apr-June,no dementia,1845,male,2008
50,home,50,12.5,1,Apr-June,no dementia,12211,male,2008
50,hospital,50,12.5,1,Apr-June,no dementia,98211,male,2008

50,elsehwere,50,12.5,1,Apr-June,no dementia,2096,male,2008
51,LTCF,51,12.75,1,July-Sept,no dementia,1782,male,2008
51,home,51,12.75,1,July-Sept,no dementia,11703,male,2008
51,hospital,51,12.75,1,July-Sept,no dementia,94043,male,2008
51,elsehwere,51,12.75,1,July-Sept,no dementia,2173,male,2008
52,LTCF,52,13,1,Oct-Dec,no dementia,2174,male,2008
52,home,52,13,1,Oct-Dec,no dementia,14801,male,2008
52,hospital,52,13,1,Oct-Dec,no dementia,107329,male,2008
52,elsehwere,52,13,1,Oct-Dec,no dementia,2485,male,2008
53,LTCF,53,13.25,1,Jan-Mar,no dementia,2428,male,2009
53,home,53,13.25,1,Jan-Mar,no dementia,16607,male,2009
53,hospital,53,13.25,1,Jan-Mar,no dementia,110844,male,2009
53,elsehwere,53,13.25,1,Jan-Mar,no dementia,2578,male,2009
54,LTCF,54,13.5,1,Apr-June,no dementia,2020,male,2009
54,home,54,13.5,1,Apr-June,no dementia,12123,male,2009
54,hospital,54,13.5,1,Apr-June,no dementia,98949,male,2009
54,elsehwere,54,13.5,1,Apr-June,no dementia,2406,male,2009
55,LTCF,55,13.75,1,July-Sept,no dementia,2076,male,2009
55,home,55,13.75,1,July-Sept,no dementia,11252,male,2009
55,hospital,55,13.75,1,July-Sept,no dementia,96320,male,2009
55,elsehwere,55,13.75,1,July-Sept,no dementia,2284,male,2009
56,LTCF,56,14,1,Oct-Dec,no dementia,2306,male,2009
56,home,56,14,1,Oct-Dec,no dementia,14571,male,2009
56,hospital,56,14,1,Oct-Dec,no dementia,107719,male,2009
56,elsehwere,56,14,1,Oct-Dec,no dementia,2731,male,2009
57,LTCF,57,14.25,1,Jan-Mar,no dementia,2505,male,2010
57,home,57,14.25,1,Jan-Mar,no dementia,16497,male,2010
57,hospital,57,14.25,1,Jan-Mar,no dementia,113220,male,2010
57,elsehwere,57,14.25,1,Jan-Mar,no dementia,2853,male,2010
58,LTCF,58,14.5,1,Apr-June,no dementia,2357,male,2010
58,home,58,14.5,1,Apr-June,no dementia,12924,male,2010
58,hospital,58,14.5,1,Apr-June,no dementia,104353,male,2010
58,elsehwere,58,14.5,1,Apr-June,no dementia,2738,male,2010
59,LTCF,59,14.75,1,July-Sept,no dementia,2253,male,2010
59,home,59,14.75,1,July-Sept,no dementia,13307,male,2010
59,hospital,59,14.75,1,July-Sept,no dementia,101330,male,2010
59,elsehwere,59,14.75,1,July-Sept,no dementia,2766,male,2010
60,LTCF,60,15,1,Oct-Dec,no dementia,2839,male,2010
60,home,60,15,1,Oct-Dec,no dementia,15749,male,2010
60,hospital,60,15,1,Oct-Dec,no dementia,113196,male,2010
60,elsehwere,60,15,1,Oct-Dec,no dementia,2942,male,2010
61,LTCF,61,15.25,1,Jan-Mar,no dementia,3161,male,2011
61,home,61,15.25,1,Jan-Mar,no dementia,18861,male,2011
61,hospital,61,15.25,1,Jan-Mar,no dementia,119800,male,2011
61,elsehwere,61,15.25,1,Jan-Mar,no dementia,7222,male,2011
62,LTCF,62,15.5,1,Apr-June,no dementia,2963,male,2011
62,home,62,15.5,1,Apr-June,no dementia,13823,male,2011
62,hospital,62,15.5,1,Apr-June,no dementia,106932,male,2011
62,elsehwere,62,15.5,1,Apr-June,no dementia,2878,male,2011
63,LTCF,63,15.75,1,July-Sept,no dementia,2630,male,2011

63,home,63,15.75,1,July-Sept,no dementia,12380,male,2011
63,hospital,63,15.75,1,July-Sept,no dementia,101222,male,2011
63,elsehwere,63,15.75,1,July-Sept,no dementia,2845,male,2011
64,LTCF,64,16,1,Oct-Dec,no dementia,3285,male,2011
64,home,64,16,1,Oct-Dec,no dementia,15880,male,2011
64,hospital,64,16,1,Oct-Dec,no dementia,113801,male,2011
64,elsehwere,64,16,1,Oct-Dec,no dementia,3149,male,2011
65,LTCF,65,16.25,1,Jan-Mar,no dementia,3587,male,2012
65,home,65,16.25,1,Jan-Mar,no dementia,19837,male,2012
65,hospital,65,16.25,1,Jan-Mar,no dementia,122952,male,2012
65,elsehwere,65,16.25,1,Jan-Mar,no dementia,3420,male,2012
66,LTCF,66,16.5,1,Apr-June,no dementia,3313,male,2012
66,home,66,16.5,1,Apr-June,no dementia,13776,male,2012
66,hospital,66,16.5,1,Apr-June,no dementia,104249,male,2012
66,elsehwere,66,16.5,1,Apr-June,no dementia,3096,male,2012
67,LTCF,67,16.75,1,July-Sept,no dementia,3264,male,2012
67,home,67,16.75,1,July-Sept,no dementia,13294,male,2012
67,hospital,67,16.75,1,July-Sept,no dementia,101220,male,2012
67,elsehwere,67,16.75,1,July-Sept,no dementia,3119,male,2012
68,LTCF,68,17,1,Oct-Dec,no dementia,4113,male,2012
68,home,68,17,1,Oct-Dec,no dementia,17708,male,2012
68,hospital,68,17,1,Oct-Dec,no dementia,118171,male,2012
68,elsehwere,68,17,1,Oct-Dec,no dementia,3693,male,2012
69,LTCF,69,17.25,1,Jan-Mar,no dementia,4312,male,2013
69,home,69,17.25,1,Jan-Mar,no dementia,19742,male,2013
69,hospital,69,17.25,1,Jan-Mar,no dementia,122831,male,2013
69,elsehwere,69,17.25,1,Jan-Mar,no dementia,3915,male,2013
70,LTCF,70,17.5,1,Apr-June,no dementia,3960,male,2013
70,home,70,17.5,1,Apr-June,no dementia,14440,male,2013
70,hospital,70,17.5,1,Apr-June,no dementia,106283,male,2013
70,elsehwere,70,17.5,1,Apr-June,no dementia,3345,male,2013
71,LTCF,71,17.75,1,July-Sept,no dementia,3722,male,2013
71,home,71,17.75,1,July-Sept,no dementia,14528,male,2013
71,hospital,71,17.75,1,July-Sept,no dementia,103420,male,2013
71,elsehwere,71,17.75,1,July-Sept,no dementia,3452,male,2013
72,LTCF,72,18,1,Oct-Dec,no dementia,4491,male,2013
72,home,72,18,1,Oct-Dec,no dementia,17755,male,2013
72,hospital,72,18,1,Oct-Dec,no dementia,116958,male,2013
72,elsehwere,72,18,1,Oct-Dec,no dementia,4006,male,2013
73,LTCF,73,18.25,1,Jan-Mar,no dementia,4499,male,2014
73,home,73,18.25,1,Jan-Mar,no dementia,20023,male,2014
73,hospital,73,18.25,1,Jan-Mar,no dementia,123380,male,2014
73,elsehwere,73,18.25,1,Jan-Mar,no dementia,4054,male,2014
74,LTCF,74,18.5,1,Apr-June,no dementia,4464,male,2014
74,home,74,18.5,1,Apr-June,no dementia,14827,male,2014
74,hospital,74,18.5,1,Apr-June,no dementia,106019,male,2014
74,elsehwere,74,18.5,1,Apr-June,no dementia,3648,male,2014
75,LTCF,75,18.75,1,July-Sept,no dementia,4358,male,2014
75,home,75,18.75,1,July-Sept,no dementia,14316,male,2014
75,hospital,75,18.75,1,July-Sept,no dementia,104531,male,2014

75,elsehwere,75,18.75,1,July-Sept,no dementia,3663,male,2014
76,LTCF,76,19,1,Oct-Dec,no dementia,5049,male,2014
76,home,76,19,1,Oct-Dec,no dementia,18410,male,2014
76,hospital,76,19,1,Oct-Dec,no dementia,119252,male,2014
76,elsehwere,76,19,1,Oct-Dec,no dementia,4136,male,2014
77,LTCF,77,19.25,1,Jan-Mar,no dementia,5338,male,2015
77,home,77,19.25,1,Jan-Mar,no dementia,20618,male,2015
77,hospital,77,19.25,1,Jan-Mar,no dementia,126625,male,2015
77,elsehwere,77,19.25,1,Jan-Mar,no dementia,4454,male,2015
78,LTCF,78,19.5,1,Apr-June,no dementia,4834,male,2015
78,home,78,19.5,1,Apr-June,no dementia,15278,male,2015
78,hospital,78,19.5,1,Apr-June,no dementia,108228,male,2015
78,elsehwere,78,19.5,1,Apr-June,no dementia,3994,male,2015
79,LTCF,79,19.75,1,July-Sept,no dementia,4790,male,2015
79,home,79,19.75,1,July-Sept,no dementia,15626,male,2015
79,hospital,79,19.75,1,July-Sept,no dementia,106530,male,2015
79,elsehwere,79,19.75,1,July-Sept,no dementia,3894,male,2015
80,LTCF,80,20,1,Oct-Dec,no dementia,5640,male,2015
80,home,80,20,1,Oct-Dec,no dementia,17836,male,2015
80,hospital,80,20,1,Oct-Dec,no dementia,118027,male,2015
80,elsehwere,80,20,1,Oct-Dec,no dementia,4429,male,2015
81,LTCF,81,20.25,1,Jan-Mar,no dementia,5748,male,2016
81,home,81,20.25,1,Jan-Mar,no dementia,20744,male,2016
81,hospital,81,20.25,1,Jan-Mar,no dementia,126062,male,2016
81,elsehwere,81,20.25,1,Jan-Mar,no dementia,4449,male,2016
82,LTCF,82,20.5,1,Apr-June,no dementia,5231,male,2016
82,home,82,20.5,1,Apr-June,no dementia,15889,male,2016
82,hospital,82,20.5,1,Apr-June,no dementia,108452,male,2016
82,elsehwere,82,20.5,1,Apr-June,no dementia,4134,male,2016
83,LTCF,83,20.75,1,July-Sept,no dementia,5379,male,2016
83,home,83,20.75,1,July-Sept,no dementia,16212,male,2016
83,hospital,83,20.75,1,July-Sept,no dementia,107355,male,2016
83,elsehwere,83,20.75,1,July-Sept,no dementia,4133,male,2016
84,LTCF,84,21,1,Oct-Dec,no dementia,6387,male,2016
84,home,84,21,1,Oct-Dec,no dementia,19554,male,2016
84,hospital,84,21,1,Oct-Dec,no dementia,122800,male,2016
84,elsehwere,84,21,1,Oct-Dec,no dementia,4626,male,2016
85,LTCF,1,.25,0,Jan-Mar,no dementia,2688,female,1996
85,home,1,.25,0,Jan-Mar,no dementia,22013,female,1996
85,hospital,1,.25,0,Jan-Mar,no dementia,68759,female,1996
85,elsehwere,1,.25,0,Jan-Mar,no dementia,1400,female,1996
86,LTCF,2,.5,0,Apr-June,no dementia,2276,female,1996
86,home,2,.5,0,Apr-June,no dementia,15460,female,1996
86,hospital,2,.5,0,Apr-June,no dementia,61569,female,1996
86,elsehwere,2,.5,0,Apr-June,no dementia,1200,female,1996
87,LTCF,3,.75,0,July-Sept,no dementia,2200,female,1996
87,home,3,.75,0,July-Sept,no dementia,13411,female,1996
87,hospital,3,.75,0,July-Sept,no dementia,58415,female,1996
87,elsehwere,3,.75,0,July-Sept,no dementia,1079,female,1996
88,LTCF,4,1,0,Oct-Dec,no dementia,2775,female,1996

88,home,4,1,0,Oct-Dec,no dementia,18290,female,1996
88,hospital,4,1,0,Oct-Dec,no dementia,65814,female,1996
88,elsehwere,4,1,0,Oct-Dec,no dementia,1390,female,1996
89,LTCF,5,1.25,0,Jan-Mar,no dementia,3287,female,1997
89,home,5,1.25,0,Jan-Mar,no dementia,21896,female,1997
89,hospital,5,1.25,0,Jan-Mar,no dementia,77518,female,1997
89,elsehwere,5,1.25,0,Jan-Mar,no dementia,1556,female,1997
90,LTCF,6,1.5,0,Apr-June,no dementia,2465,female,1997
90,home,6,1.5,0,Apr-June,no dementia,15046,female,1997
90,hospital,6,1.5,0,Apr-June,no dementia,62500,female,1997
90,elsehwere,6,1.5,0,Apr-June,no dementia,1241,female,1997
91,LTCF,7,1.75,0,July-Sept,no dementia,2082,female,1997
91,home,7,1.75,0,July-Sept,no dementia,12796,female,1997
91,hospital,7,1.75,0,July-Sept,no dementia,58224,female,1997
91,elsehwere,7,1.75,0,July-Sept,no dementia,1157,female,1997
92,LTCF,8,2,0,Oct-Dec,no dementia,2723,female,1997
92,home,8,2,0,Oct-Dec,no dementia,16833,female,1997
92,hospital,8,2,0,Oct-Dec,no dementia,66566,female,1997
92,elsehwere,8,2,0,Oct-Dec,no dementia,1430,female,1997
93,LTCF,9,2.25,0,Jan-Mar,no dementia,3281,female,1998
93,home,9,2.25,0,Jan-Mar,no dementia,21080,female,1998
93,hospital,9,2.25,0,Jan-Mar,no dementia,77339,female,1998
93,elsehwere,9,2.25,0,Jan-Mar,no dementia,1657,female,1998
94,LTCF,10,2.5,0,Apr-June,no dementia,2505,female,1998
94,home,10,2.5,0,Apr-June,no dementia,14232,female,1998
94,hospital,10,2.5,0,Apr-June,no dementia,63880,female,1998
94,elsehwere,10,2.5,0,Apr-June,no dementia,1380,female,1998
95,LTCF,11,2.75,0,July-Sept,no dementia,2385,female,1998
95,home,11,2.75,0,July-Sept,no dementia,13222,female,1998
95,hospital,11,2.75,0,July-Sept,no dementia,62607,female,1998
95,elsehwere,11,2.75,0,July-Sept,no dementia,1313,female,1998
96,LTCF,12,3,0,Oct-Dec,no dementia,2794,female,1998
96,home,12,3,0,Oct-Dec,no dementia,16763,female,1998
96,hospital,12,3,0,Oct-Dec,no dementia,69937,female,1998
96,elsehwere,12,3,0,Oct-Dec,no dementia,1576,female,1998
97,LTCF,13,3.25,0,Jan-Mar,no dementia,3678,female,1999
97,home,13,3.25,0,Jan-Mar,no dementia,22355,female,1999
97,hospital,13,3.25,0,Jan-Mar,no dementia,91599,female,1999
97,elsehwere,13,3.25,0,Jan-Mar,no dementia,1968,female,1999
98,LTCF,14,3.5,0,Apr-June,no dementia,2712,female,1999
98,home,14,3.5,0,Apr-June,no dementia,13832,female,1999
98,hospital,14,3.5,0,Apr-June,no dementia,68495,female,1999
98,elsehwere,14,3.5,0,Apr-June,no dementia,1472,female,1999
99,LTCF,15,3.75,0,July-Sept,no dementia,2364,female,1999
99,home,15,3.75,0,July-Sept,no dementia,12027,female,1999
99,hospital,15,3.75,0,July-Sept,no dementia,64195,female,1999
99,elsehwere,15,3.75,0,July-Sept,no dementia,1280,female,1999
100,LTCF,16,4,0,Oct-Dec,no dementia,2963,female,1999
100,home,16,4,0,Oct-Dec,no dementia,15695,female,1999
100,hospital,16,4,0,Oct-Dec,no dementia,72497,female,1999

100,elsehwere,16,4,0,Oct-Dec,no dementia,1534,female,1999
101,LTCF,17,4.25,0,Jan-Mar,no dementia,3571,female,2000
101,home,17,4.25,0,Jan-Mar,no dementia,19495,female,2000
101,hospital,17,4.25,0,Jan-Mar,no dementia,85915,female,2000
101,elsehwere,17,4.25,0,Jan-Mar,no dementia,1776,female,2000
102,LTCF,18,4.5,0,Apr-June,no dementia,3046,female,2000
102,home,18,4.5,0,Apr-June,no dementia,11908,female,2000
102,hospital,18,4.5,0,Apr-June,no dementia,69724,female,2000
102,elsehwere,18,4.5,0,Apr-June,no dementia,1512,female,2000
103,LTCF,19,4.75,0,July-Sept,no dementia,2679,female,2000
103,home,19,4.75,0,July-Sept,no dementia,10684,female,2000
103,hospital,19,4.75,0,July-Sept,no dementia,65150,female,2000
103,elsehwere,19,4.75,0,July-Sept,no dementia,1371,female,2000
104,LTCF,20,5,0,Oct-Dec,no dementia,3332,female,2000
104,home,20,5,0,Oct-Dec,no dementia,14310,female,2000
104,hospital,20,5,0,Oct-Dec,no dementia,73176,female,2000
104,elsehwere,20,5,0,Oct-Dec,no dementia,1697,female,2000
105,LTCF,21,5.25,0,Jan-Mar,no dementia,3678,female,2001
105,home,21,5.25,0,Jan-Mar,no dementia,16821,female,2001
105,hospital,21,5.25,0,Jan-Mar,no dementia,81225,female,2001
105,elsehwere,21,5.25,0,Jan-Mar,no dementia,1819,female,2001
106,LTCF,22,5.5,0,Apr-June,no dementia,3129,female,2001
106,home,22,5.5,0,Apr-June,no dementia,12139,female,2001
106,hospital,22,5.5,0,Apr-June,no dementia,73424,female,2001
106,elsehwere,22,5.5,0,Apr-June,no dementia,1607,female,2001
107,LTCF,23,5.75,0,July-Sept,no dementia,2974,female,2001
107,home,23,5.75,0,July-Sept,no dementia,11091,female,2001
107,hospital,23,5.75,0,July-Sept,no dementia,68843,female,2001
107,elsehwere,23,5.75,0,July-Sept,no dementia,1491,female,2001
108,LTCF,24,6,0,Oct-Dec,no dementia,3659,female,2001
108,home,24,6,0,Oct-Dec,no dementia,14600,female,2001
108,hospital,24,6,0,Oct-Dec,no dementia,78243,female,2001
108,elsehwere,24,6,0,Oct-Dec,no dementia,1897,female,2001
109,LTCF,25,6.25,0,Jan-Mar,no dementia,3680,female,2002
109,home,25,6.25,0,Jan-Mar,no dementia,16068,female,2002
109,hospital,25,6.25,0,Jan-Mar,no dementia,83197,female,2002
109,elsehwere,25,6.25,0,Jan-Mar,no dementia,1864,female,2002
110,LTCF,26,6.5,0,Apr-June,no dementia,3092,female,2002
110,home,26,6.5,0,Apr-June,no dementia,11827,female,2002
110,hospital,26,6.5,0,Apr-June,no dementia,73620,female,2002
110,elsehwere,26,6.5,0,Apr-June,no dementia,1644,female,2002
111,LTCF,27,6.75,0,July-Sept,no dementia,3058,female,2002
111,home,27,6.75,0,July-Sept,no dementia,11371,female,2002
111,hospital,27,6.75,0,July-Sept,no dementia,70458,female,2002
111,elsehwere,27,6.75,0,July-Sept,no dementia,1618,female,2002
112,LTCF,28,7,0,Oct-Dec,no dementia,3568,female,2002
112,home,28,7,0,Oct-Dec,no dementia,14598,female,2002
112,hospital,28,7,0,Oct-Dec,no dementia,81651,female,2002
112,elsehwere,28,7,0,Oct-Dec,no dementia,1877,female,2002
113,LTCF,29,7.25,0,Jan-Mar,no dementia,4138,female,2003

113,home,29,7.25,0,Jan-Mar,no dementia,17664,female,2003
113,hospital,29,7.25,0,Jan-Mar,no dementia,92790,female,2003
113,elsehwere,29,7.25,0,Jan-Mar,no dementia,2156,female,2003
114,LTCF,30,7.5,0,Apr-June,no dementia,3361,female,2003
114,home,30,7.5,0,Apr-June,no dementia,11880,female,2003
114,hospital,30,7.5,0,Apr-June,no dementia,76990,female,2003
114,elsehwere,30,7.5,0,Apr-June,no dementia,1826,female,2003
115,LTCF,31,7.75,0,July-Sept,no dementia,3053,female,2003
115,home,31,7.75,0,July-Sept,no dementia,10342,female,2003
115,hospital,31,7.75,0,July-Sept,no dementia,72989,female,2003
115,elsehwere,31,7.75,0,July-Sept,no dementia,1599,female,2003
116,LTCF,32,8,0,Oct-Dec,no dementia,3603,female,2003
116,home,32,8,0,Oct-Dec,no dementia,13523,female,2003
116,hospital,32,8,0,Oct-Dec,no dementia,82222,female,2003
116,elsehwere,32,8,0,Oct-Dec,no dementia,1853,female,2003
117,LTCF,33,8.25,0,Jan-Mar,no dementia,4321,female,2004
117,home,33,8.25,0,Jan-Mar,no dementia,16132,female,2004
117,hospital,33,8.25,0,Jan-Mar,no dementia,91716,female,2004
117,elsehwere,33,8.25,0,Jan-Mar,no dementia,2203,female,2004
118,LTCF,34,8.5,0,Apr-June,no dementia,3573,female,2004
118,home,34,8.5,0,Apr-June,no dementia,11131,female,2004
118,hospital,34,8.5,0,Apr-June,no dementia,78480,female,2004
118,elsehwere,34,8.5,0,Apr-June,no dementia,1821,female,2004
119,LTCF,35,8.75,0,July-Sept,no dementia,3302,female,2004
119,home,35,8.75,0,July-Sept,no dementia,10179,female,2004
119,hospital,35,8.75,0,July-Sept,no dementia,76160,female,2004
119,elsehwere,35,8.75,0,July-Sept,no dementia,1801,female,2004
120,LTCF,36,9,0,Oct-Dec,no dementia,4154,female,2004
120,home,36,9,0,Oct-Dec,no dementia,12968,female,2004
120,hospital,36,9,0,Oct-Dec,no dementia,87265,female,2004
120,elsehwere,36,9,0,Oct-Dec,no dementia,2064,female,2004
121,LTCF,37,9.25,0,Jan-Mar,no dementia,4612,female,2005
121,home,37,9.25,0,Jan-Mar,no dementia,17087,female,2005
121,hospital,37,9.25,0,Jan-Mar,no dementia,100932,female,2005
121,elsehwere,37,9.25,0,Jan-Mar,no dementia,2492,female,2005
122,LTCF,38,9.5,0,Apr-June,no dementia,3872,female,2005
122,home,38,9.5,0,Apr-June,no dementia,11495,female,2005
122,hospital,38,9.5,0,Apr-June,no dementia,84707,female,2005
122,elsehwere,38,9.5,0,Apr-June,no dementia,2035,female,2005
123,LTCF,39,9.75,0,July-Sept,no dementia,3734,female,2005
123,home,39,9.75,0,July-Sept,no dementia,9846,female,2005
123,hospital,39,9.75,0,July-Sept,no dementia,79668,female,2005
123,elsehwere,39,9.75,0,July-Sept,no dementia,1905,female,2005
124,LTCF,40,10,0,Oct-Dec,no dementia,4425,female,2005
124,home,40,10,0,Oct-Dec,no dementia,14137,female,2005
124,hospital,40,10,0,Oct-Dec,no dementia,91451,female,2005
124,elsehwere,40,10,0,Oct-Dec,no dementia,2218,female,2005
125,LTCF,41,10.25,0,Jan-Mar,no dementia,4943,female,2006
125,home,41,10.25,0,Jan-Mar,no dementia,16101,female,2006
125,hospital,41,10.25,0,Jan-Mar,no dementia,98926,female,2006

125,elsehwere,41,10.25,0,Jan-Mar,no dementia,2521,female,2006
126,LTCF,42,10.5,1,Apr-June,no dementia,4393,female,2006
126,home,42,10.5,1,Apr-June,no dementia,11708,female,2006
126,hospital,42,10.5,1,Apr-June,no dementia,87406,female,2006
126,elsehwere,42,10.5,1,Apr-June,no dementia,2283,female,2006
127,LTCF,43,10.75,1,July-Sept,no dementia,4023,female,2006
127,home,43,10.75,1,July-Sept,no dementia,10383,female,2006
127,hospital,43,10.75,1,July-Sept,no dementia,81939,female,2006
127,elsehwere,43,10.75,1,July-Sept,no dementia,2171,female,2006
128,LTCF,44,11,1,Oct-Dec,no dementia,4907,female,2006
128,home,44,11,1,Oct-Dec,no dementia,13677,female,2006
128,hospital,44,11,1,Oct-Dec,no dementia,92270,female,2006
128,elsehwere,44,11,1,Oct-Dec,no dementia,2531,female,2006
129,LTCF,45,11.25,1,Jan-Mar,no dementia,5234,female,2007
129,home,45,11.25,1,Jan-Mar,no dementia,15492,female,2007
129,hospital,45,11.25,1,Jan-Mar,no dementia,99852,female,2007
129,elsehwere,45,11.25,1,Jan-Mar,no dementia,2738,female,2007
130,LTCF,46,11.5,1,Apr-June,no dementia,4837,female,2007
130,home,46,11.5,1,Apr-June,no dementia,11898,female,2007
130,hospital,46,11.5,1,Apr-June,no dementia,88679,female,2007
130,elsehwere,46,11.5,1,Apr-June,no dementia,2446,female,2007
131,LTCF,47,11.75,1,July-Sept,no dementia,4567,female,2007
131,home,47,11.75,1,July-Sept,no dementia,11481,female,2007
131,hospital,47,11.75,1,July-Sept,no dementia,84496,female,2007
131,elsehwere,47,11.75,1,July-Sept,no dementia,2467,female,2007
132,LTCF,48,12,1,Oct-Dec,no dementia,5509,female,2007
132,home,48,12,1,Oct-Dec,no dementia,14258,female,2007
132,hospital,48,12,1,Oct-Dec,no dementia,96258,female,2007
132,elsehwere,48,12,1,Oct-Dec,no dementia,2872,female,2007
133,LTCF,49,12.25,1,Jan-Mar,no dementia,6193,female,2008
133,home,49,12.25,1,Jan-Mar,no dementia,17685,female,2008
133,hospital,49,12.25,1,Jan-Mar,no dementia,104619,female,2008
133,elsehwere,49,12.25,1,Jan-Mar,no dementia,3163,female,2008
134,LTCF,50,12.5,1,Apr-June,no dementia,5512,female,2008
134,home,50,12.5,1,Apr-June,no dementia,12394,female,2008
134,hospital,50,12.5,1,Apr-June,no dementia,89888,female,2008
134,elsehwere,50,12.5,1,Apr-June,no dementia,2885,female,2008
135,LTCF,51,12.75,1,July-Sept,no dementia,5328,female,2008
135,home,51,12.75,1,July-Sept,no dementia,11576,female,2008
135,hospital,51,12.75,1,July-Sept,no dementia,86869,female,2008
135,elsehwere,51,12.75,1,July-Sept,no dementia,2806,female,2008
136,LTCF,52,13,1,Oct-Dec,no dementia,6451,female,2008
136,home,52,13,1,Oct-Dec,no dementia,14654,female,2008
136,hospital,52,13,1,Oct-Dec,no dementia,97927,female,2008
136,elsehwere,52,13,1,Oct-Dec,no dementia,3294,female,2008
137,LTCF,53,13.25,1,Jan-Mar,no dementia,6799,female,2009
137,home,53,13.25,1,Jan-Mar,no dementia,16638,female,2009
137,hospital,53,13.25,1,Jan-Mar,no dementia,101749,female,2009
137,elsehwere,53,13.25,1,Jan-Mar,no dementia,3451,female,2009
138,LTCF,54,13.5,1,Apr-June,no dementia,6023,female,2009

138,home,54,13.5,1,Apr-June,no dementia,12022,female,2009
138,hospital,54,13.5,1,Apr-June,no dementia,89882,female,2009
138,elsehwere,54,13.5,1,Apr-June,no dementia,3274,female,2009
139,LTCF,55,13.75,1,July-Sept,no dementia,6020,female,2009
139,home,55,13.75,1,July-Sept,no dementia,10828,female,2009
139,hospital,55,13.75,1,July-Sept,no dementia,87819,female,2009
139,elsehwere,55,13.75,1,July-Sept,no dementia,3156,female,2009
140,LTCF,56,14,1,Oct-Dec,no dementia,7053,female,2009
140,home,56,14,1,Oct-Dec,no dementia,14742,female,2009
140,hospital,56,14,1,Oct-Dec,no dementia,98829,female,2009
140,elsehwere,56,14,1,Oct-Dec,no dementia,3701,female,2009
141,LTCF,57,14.25,1,Jan-Mar,no dementia,7207,female,2010
141,home,57,14.25,1,Jan-Mar,no dementia,16943,female,2010
141,hospital,57,14.25,1,Jan-Mar,no dementia,103570,female,2010
141,elsehwere,57,14.25,1,Jan-Mar,no dementia,3897,female,2010
142,LTCF,58,14.5,1,Apr-June,no dementia,6927,female,2010
142,home,58,14.5,1,Apr-June,no dementia,12850,female,2010
142,hospital,58,14.5,1,Apr-June,no dementia,96022,female,2010
142,elsehwere,58,14.5,1,Apr-June,no dementia,3924,female,2010
143,LTCF,59,14.75,1,July-Sept,no dementia,7107,female,2010
143,home,59,14.75,1,July-Sept,no dementia,12611,female,2010
143,hospital,59,14.75,1,July-Sept,no dementia,94091,female,2010
143,elsehwere,59,14.75,1,July-Sept,no dementia,3868,female,2010
144,LTCF,60,15,1,Oct-Dec,no dementia,8288,female,2010
144,home,60,15,1,Oct-Dec,no dementia,15710,female,2010
144,hospital,60,15,1,Oct-Dec,no dementia,103326,female,2010
144,elsehwere,60,15,1,Oct-Dec,no dementia,4391,female,2010
145,LTCF,61,15.25,1,Jan-Mar,no dementia,8934,female,2011
145,home,61,15.25,1,Jan-Mar,no dementia,18887,female,2011
145,hospital,61,15.25,1,Jan-Mar,no dementia,110445,female,2011
145,elsehwere,61,15.25,1,Jan-Mar,no dementia,9967,female,2011
146,LTCF,62,15.5,1,Apr-June,no dementia,8368,female,2011
146,home,62,15.5,1,Apr-June,no dementia,13655,female,2011
146,hospital,62,15.5,1,Apr-June,no dementia,99263,female,2011
146,elsehwere,62,15.5,1,Apr-June,no dementia,4489,female,2011
147,LTCF,63,15.75,1,July-Sept,no dementia,7858,female,2011
147,home,63,15.75,1,July-Sept,no dementia,11754,female,2011
147,hospital,63,15.75,1,July-Sept,no dementia,94587,female,2011
147,elsehwere,63,15.75,1,July-Sept,no dementia,4310,female,2011
148,LTCF,64,16,1,Oct-Dec,no dementia,9311,female,2011
148,home,64,16,1,Oct-Dec,no dementia,15182,female,2011
148,hospital,64,16,1,Oct-Dec,no dementia,104667,female,2011
148,elsehwere,64,16,1,Oct-Dec,no dementia,4880,female,2011
149,LTCF,65,16.25,1,Jan-Mar,no dementia,10126,female,2012
149,home,65,16.25,1,Jan-Mar,no dementia,19461,female,2012
149,hospital,65,16.25,1,Jan-Mar,no dementia,114725,female,2012
149,elsehwere,65,16.25,1,Jan-Mar,no dementia,5444,female,2012
150,LTCF,66,16.5,1,Apr-June,no dementia,8989,female,2012
150,home,66,16.5,1,Apr-June,no dementia,13316,female,2012
150,hospital,66,16.5,1,Apr-June,no dementia,97194,female,2012

150,elsehwere,66,16.5,1,Apr-June,no dementia,5014,female,2012
151,LTCF,67,16.75,1,July-Sept,no dementia,9344,female,2012
151,home,67,16.75,1,July-Sept,no dementia,12548,female,2012
151,hospital,67,16.75,1,July-Sept,no dementia,93764,female,2012
151,elsehwere,67,16.75,1,July-Sept,no dementia,4977,female,2012
152,LTCF,68,17,1,Oct-Dec,no dementia,11217,female,2012
152,home,68,17,1,Oct-Dec,no dementia,16741,female,2012
152,hospital,68,17,1,Oct-Dec,no dementia,108084,female,2012
152,elsehwere,68,17,1,Oct-Dec,no dementia,5763,female,2012
153,LTCF,69,17.25,1,Jan-Mar,no dementia,11743,female,2013
153,home,69,17.25,1,Jan-Mar,no dementia,19072,female,2013
153,hospital,69,17.25,1,Jan-Mar,no dementia,114202,female,2013
153,elsehwere,69,17.25,1,Jan-Mar,no dementia,6033,female,2013
154,LTCF,70,17.5,1,Apr-June,no dementia,10479,female,2013
154,home,70,17.5,1,Apr-June,no dementia,13674,female,2013
154,hospital,70,17.5,1,Apr-June,no dementia,98027,female,2013
154,elsehwere,70,17.5,1,Apr-June,no dementia,5451,female,2013
155,LTCF,71,17.75,1,July-Sept,no dementia,10560,female,2013
155,home,71,17.75,1,July-Sept,no dementia,13438,female,2013
155,hospital,71,17.75,1,July-Sept,no dementia,95398,female,2013
155,elsehwere,71,17.75,1,July-Sept,no dementia,5515,female,2013
156,LTCF,72,18,1,Oct-Dec,no dementia,12315,female,2013
156,home,72,18,1,Oct-Dec,no dementia,16492,female,2013
156,hospital,72,18,1,Oct-Dec,no dementia,106941,female,2013
156,elsehwere,72,18,1,Oct-Dec,no dementia,6261,female,2013
157,LTCF,73,18.25,1,Jan-Mar,no dementia,12654,female,2014
157,home,73,18.25,1,Jan-Mar,no dementia,18961,female,2014
157,hospital,73,18.25,1,Jan-Mar,no dementia,112903,female,2014
157,elsehwere,73,18.25,1,Jan-Mar,no dementia,6390,female,2014
158,LTCF,74,18.5,1,Apr-June,no dementia,11338,female,2014
158,home,74,18.5,1,Apr-June,no dementia,13700,female,2014
158,hospital,74,18.5,1,Apr-June,no dementia,97328,female,2014
158,elsehwere,74,18.5,1,Apr-June,no dementia,5914,female,2014
159,LTCF,75,18.75,1,July-Sept,no dementia,11557,female,2014
159,home,75,18.75,1,July-Sept,no dementia,12689,female,2014
159,hospital,75,18.75,1,July-Sept,no dementia,94650,female,2014
159,elsehwere,75,18.75,1,July-Sept,no dementia,5876,female,2014
160,LTCF,76,19,1,Oct-Dec,no dementia,13366,female,2014
160,home,76,19,1,Oct-Dec,no dementia,16933,female,2014
160,hospital,76,19,1,Oct-Dec,no dementia,108387,female,2014
160,elsehwere,76,19,1,Oct-Dec,no dementia,6601,female,2014
161,LTCF,77,19.25,1,Jan-Mar,no dementia,14286,female,2015
161,home,77,19.25,1,Jan-Mar,no dementia,19357,female,2015
161,hospital,77,19.25,1,Jan-Mar,no dementia,116626,female,2015
161,elsehwere,77,19.25,1,Jan-Mar,no dementia,7296,female,2015
162,LTCF,78,19.5,1,Apr-June,no dementia,12570,female,2015
162,home,78,19.5,1,Apr-June,no dementia,13597,female,2015
162,hospital,78,19.5,1,Apr-June,no dementia,98396,female,2015
162,elsehwere,78,19.5,1,Apr-June,no dementia,6309,female,2015
163,LTCF,79,19.75,1,July-Sept,no dementia,12625,female,2015

163,home,79,19.75,1,July-Sept,no dementia,13569,female,2015
163,hospital,79,19.75,1,July-Sept,no dementia,96414,female,2015
163,elsehwere,79,19.75,1,July-Sept,no dementia,6274,female,2015
164,LTCF,80,20,1,Oct-Dec,no dementia,14753,female,2015
164,home,80,20,1,Oct-Dec,no dementia,16385,female,2015
164,hospital,80,20,1,Oct-Dec,no dementia,106464,female,2015
164,elsehwere,80,20,1,Oct-Dec,no dementia,7118,female,2015
165,LTCF,81,20.25,1,Jan-Mar,no dementia,15137,female,2016
165,home,81,20.25,1,Jan-Mar,no dementia,19210,female,2016
165,hospital,81,20.25,1,Jan-Mar,no dementia,113064,female,2016
165,elsehwere,81,20.25,1,Jan-Mar,no dementia,7442,female,2016
166,LTCF,82,20.5,1,Apr-June,no dementia,13701,female,2016
166,home,82,20.5,1,Apr-June,no dementia,14417,female,2016
166,hospital,82,20.5,1,Apr-June,no dementia,97581,female,2016
166,elsehwere,82,20.5,1,Apr-June,no dementia,6420,female,2016
167,LTCF,83,20.75,1,July-Sept,no dementia,14033,female,2016
167,home,83,20.75,1,July-Sept,no dementia,14034,female,2016
167,hospital,83,20.75,1,July-Sept,no dementia,97189,female,2016
167,elsehwere,83,20.75,1,July-Sept,no dementia,6510,female,2016
168,LTCF,84,21,1,Oct-Dec,no dementia,16467,female,2016
168,home,84,21,1,Oct-Dec,no dementia,17711,female,2016
168,hospital,84,21,1,Oct-Dec,no dementia,110725,female,2016
168,elsehwere,84,21,1,Oct-Dec,no dementia,7405,female,2016
169,LTCF,1,.25,0,Jan-Mar,dementia,14,male,1996
169,home,1,.25,0,Jan-Mar,dementia,111,male,1996
169,hospital,1,.25,0,Jan-Mar,dementia,203,male,1996
169,elsehwere,1,.25,0,Jan-Mar,dementia,10,male,1996
170,LTCF,2,.5,0,Apr-June,dementia,14,male,1996
170,home,2,.5,0,Apr-June,dementia,73,male,1996
170,hospital,2,.5,0,Apr-June,dementia,134,male,1996
170,elsehwere,2,.5,0,Apr-June,dementia,9,male,1996
171,LTCF,3,.75,0,July-Sept,dementia,11,male,1996
171,home,3,.75,0,July-Sept,dementia,63,male,1996
171,hospital,3,.75,0,July-Sept,dementia,141,male,1996
171,elsehwere,3,.75,0,July-Sept,dementia,8,male,1996
172,LTCF,4,1,0,Oct-Dec,dementia,15,male,1996
172,home,4,1,0,Oct-Dec,dementia,102,male,1996
172,hospital,4,1,0,Oct-Dec,dementia,167,male,1996
172,elsehwere,4,1,0,Oct-Dec,dementia,8,male,1996
173,LTCF,5,1.25,0,Jan-Mar,dementia,22,male,1997
173,home,5,1.25,0,Jan-Mar,dementia,96,male,1997
173,hospital,5,1.25,0,Jan-Mar,dementia,174,male,1997
173,elsehwere,5,1.25,0,Jan-Mar,dementia,7,male,1997
174,LTCF,6,1.5,0,Apr-June,dementia,10,male,1997
174,home,6,1.5,0,Apr-June,dementia,58,male,1997
174,hospital,6,1.5,0,Apr-June,dementia,166,male,1997
174,elsehwere,6,1.5,0,Apr-June,dementia,6,male,1997
175,LTCF,7,1.75,0,July-Sept,dementia,14,male,1997
175,home,7,1.75,0,July-Sept,dementia,59,male,1997
175,hospital,7,1.75,0,July-Sept,dementia,126,male,1997

175,elsehwere,7,1.75,0,July-Sept,dementia,9,male,1997
176,LTCF,8,2,0,Oct-Dec,dementia,21,male,1997
176,home,8,2,0,Oct-Dec,dementia,73,male,1997
176,hospital,8,2,0,Oct-Dec,dementia,174,male,1997
176,elsehwere,8,2,0,Oct-Dec,dementia,12,male,1997
177,LTCF,9,2.25,0,Jan-Mar,dementia,19,male,1998
177,home,9,2.25,0,Jan-Mar,dementia,106,male,1998
177,hospital,9,2.25,0,Jan-Mar,dementia,184,male,1998
177,elsehwere,9,2.25,0,Jan-Mar,dementia,12,male,1998
178,LTCF,10,2.5,0,Apr-June,dementia,19,male,1998
178,home,10,2.5,0,Apr-June,dementia,65,male,1998
178,hospital,10,2.5,0,Apr-June,dementia,151,male,1998
178,elsehwere,10,2.5,0,Apr-June,dementia,5,male,1998
179,LTCF,11,2.75,0,July-Sept,dementia,11,male,1998
179,home,11,2.75,0,July-Sept,dementia,45,male,1998
179,hospital,11,2.75,0,July-Sept,dementia,141,male,1998
179,elsehwere,11,2.75,0,July-Sept,dementia,9,male,1998
180,LTCF,12,3,0,Oct-Dec,dementia,25,male,1998
180,home,12,3,0,Oct-Dec,dementia,91,male,1998
180,hospital,12,3,0,Oct-Dec,dementia,213,male,1998
180,elsehwere,12,3,0,Oct-Dec,dementia,17,male,1998
181,LTCF,13,3.25,0,Jan-Mar,dementia,28,male,1999
181,home,13,3.25,0,Jan-Mar,dementia,102,male,1999
181,hospital,13,3.25,0,Jan-Mar,dementia,262,male,1999
181,elsehwere,13,3.25,0,Jan-Mar,dementia,18,male,1999
182,LTCF,14,3.5,0,Apr-June,dementia,18,male,1999
182,home,14,3.5,0,Apr-June,dementia,59,male,1999
182,hospital,14,3.5,0,Apr-June,dementia,178,male,1999
182,elsehwere,14,3.5,0,Apr-June,dementia,9,male,1999
183,LTCF,15,3.75,0,July-Sept,dementia,22,male,1999
183,home,15,3.75,0,July-Sept,dementia,51,male,1999
183,hospital,15,3.75,0,July-Sept,dementia,159,male,1999
183,elsehwere,15,3.75,0,July-Sept,dementia,13,male,1999
184,LTCF,16,4,0,Oct-Dec,dementia,21,male,1999
184,home,16,4,0,Oct-Dec,dementia,73,male,1999
184,hospital,16,4,0,Oct-Dec,dementia,213,male,1999
184,elsehwere,16,4,0,Oct-Dec,dementia,15,male,1999
185,LTCF,17,4.25,0,Jan-Mar,dementia,37,male,2000
185,home,17,4.25,0,Jan-Mar,dementia,114,male,2000
185,hospital,17,4.25,0,Jan-Mar,dementia,241,male,2000
185,elsehwere,17,4.25,0,Jan-Mar,dementia,16,male,2000
186,LTCF,18,4.5,0,Apr-June,dementia,31,male,2000
186,home,18,4.5,0,Apr-June,dementia,67,male,2000
186,hospital,18,4.5,0,Apr-June,dementia,214,male,2000
186,elsehwere,18,4.5,0,Apr-June,dementia,18,male,2000
187,LTCF,19,4.75,0,July-Sept,dementia,16,male,2000
187,home,19,4.75,0,July-Sept,dementia,59,male,2000
187,hospital,19,4.75,0,July-Sept,dementia,198,male,2000
187,elsehwere,19,4.75,0,July-Sept,dementia,16,male,2000
188,LTCF,20,5,0,Oct-Dec,dementia,27,male,2000

188,home,20,5,0,Oct-Dec,dementia,84,male,2000
188,hospital,20,5,0,Oct-Dec,dementia,272,male,2000
188,elsehwere,20,5,0,Oct-Dec,dementia,27,male,2000
189,LTCF,21,5.25,0,Jan-Mar,dementia,38,male,2001
189,home,21,5.25,0,Jan-Mar,dementia,74,male,2001
189,hospital,21,5.25,0,Jan-Mar,dementia,316,male,2001
189,elsehwere,21,5.25,0,Jan-Mar,dementia,17,male,2001
190,LTCF,22,5.5,0,Apr-June,dementia,27,male,2001
190,home,22,5.5,0,Apr-June,dementia,74,male,2001
190,hospital,22,5.5,0,Apr-June,dementia,241,male,2001
190,elsehwere,22,5.5,0,Apr-June,dementia,23,male,2001
191,LTCF,23,5.75,0,July-Sept,dementia,28,male,2001
191,home,23,5.75,0,July-Sept,dementia,59,male,2001
191,hospital,23,5.75,0,July-Sept,dementia,229,male,2001
191,elsehwere,23,5.75,0,July-Sept,dementia,16,male,2001
192,LTCF,24,6,0,Oct-Dec,dementia,33,male,2001
192,home,24,6,0,Oct-Dec,dementia,78,male,2001
192,hospital,24,6,0,Oct-Dec,dementia,262,male,2001
192,elsehwere,24,6,0,Oct-Dec,dementia,34,male,2001
193,LTCF,25,6.25,0,Jan-Mar,dementia,50,male,2002
193,home,25,6.25,0,Jan-Mar,dementia,94,male,2002
193,hospital,25,6.25,0,Jan-Mar,dementia,307,male,2002
193,elsehwere,25,6.25,0,Jan-Mar,dementia,22,male,2002
194,LTCF,26,6.5,0,Apr-June,dementia,24,male,2002
194,home,26,6.5,0,Apr-June,dementia,53,male,2002
194,hospital,26,6.5,0,Apr-June,dementia,284,male,2002
194,elsehwere,26,6.5,0,Apr-June,dementia,15,male,2002
195,LTCF,27,6.75,0,July-Sept,dementia,27,male,2002
195,home,27,6.75,0,July-Sept,dementia,57,male,2002
195,hospital,27,6.75,0,July-Sept,dementia,237,male,2002
195,elsehwere,27,6.75,0,July-Sept,dementia,15,male,2002
196,LTCF,28,7,0,Oct-Dec,dementia,32,male,2002
196,home,28,7,0,Oct-Dec,dementia,87,male,2002
196,hospital,28,7,0,Oct-Dec,dementia,297,male,2002
196,elsehwere,28,7,0,Oct-Dec,dementia,17,male,2002
197,LTCF,29,7.25,0,Jan-Mar,dementia,30,male,2003
197,home,29,7.25,0,Jan-Mar,dementia,88,male,2003
197,hospital,29,7.25,0,Jan-Mar,dementia,337,male,2003
197,elsehwere,29,7.25,0,Jan-Mar,dementia,33,male,2003
198,LTCF,30,7.5,0,Apr-June,dementia,27,male,2003
198,home,30,7.5,0,Apr-June,dementia,72,male,2003
198,hospital,30,7.5,0,Apr-June,dementia,314,male,2003
198,elsehwere,30,7.5,0,Apr-June,dementia,25,male,2003
199,LTCF,31,7.75,0,July-Sept,dementia,24,male,2003
199,home,31,7.75,0,July-Sept,dementia,60,male,2003
199,hospital,31,7.75,0,July-Sept,dementia,295,male,2003
199,elsehwere,31,7.75,0,July-Sept,dementia,17,male,2003
200,LTCF,32,8,0,Oct-Dec,dementia,37,male,2003
200,home,32,8,0,Oct-Dec,dementia,76,male,2003
200,hospital,32,8,0,Oct-Dec,dementia,324,male,2003

200,elsehwere,32,8,0,Oct-Dec,dementia,22,male,2003
201,LTCF,33,8.25,0,Jan-Mar,dementia,29,male,2004
201,home,33,8.25,0,Jan-Mar,dementia,90,male,2004
201,hospital,33,8.25,0,Jan-Mar,dementia,329,male,2004
201,elsehwere,33,8.25,0,Jan-Mar,dementia,21,male,2004
202,LTCF,34,8.5,0,Apr-June,dementia,23,male,2004
202,home,34,8.5,0,Apr-June,dementia,56,male,2004
202,hospital,34,8.5,0,Apr-June,dementia,324,male,2004
202,elsehwere,34,8.5,0,Apr-June,dementia,20,male,2004
203,LTCF,35,8.75,0,July-Sept,dementia,39,male,2004
203,home,35,8.75,0,July-Sept,dementia,52,male,2004
203,hospital,35,8.75,0,July-Sept,dementia,304,male,2004
203,elsehwere,35,8.75,0,July-Sept,dementia,28,male,2004
204,LTCF,36,9,0,Oct-Dec,dementia,31,male,2004
204,home,36,9,0,Oct-Dec,dementia,87,male,2004
204,hospital,36,9,0,Oct-Dec,dementia,375,male,2004
204,elsehwere,36,9,0,Oct-Dec,dementia,26,male,2004
205,LTCF,37,9.25,0,Jan-Mar,dementia,46,male,2005
205,home,37,9.25,0,Jan-Mar,dementia,89,male,2005
205,hospital,37,9.25,0,Jan-Mar,dementia,365,male,2005
205,elsehwere,37,9.25,0,Jan-Mar,dementia,36,male,2005
206,LTCF,38,9.5,0,Apr-June,dementia,28,male,2005
206,home,38,9.5,0,Apr-June,dementia,65,male,2005
206,hospital,38,9.5,0,Apr-June,dementia,338,male,2005
206,elsehwere,38,9.5,0,Apr-June,dementia,24,male,2005
207,LTCF,39,9.75,0,July-Sept,dementia,34,male,2005
207,home,39,9.75,0,July-Sept,dementia,47,male,2005
207,hospital,39,9.75,0,July-Sept,dementia,334,male,2005
207,elsehwere,39,9.75,0,July-Sept,dementia,19,male,2005
208,LTCF,40,10,0,Oct-Dec,dementia,29,male,2005
208,home,40,10,0,Oct-Dec,dementia,79,male,2005
208,hospital,40,10,0,Oct-Dec,dementia,381,male,2005
208,elsehwere,40,10,0,Oct-Dec,dementia,32,male,2005
209,LTCF,41,10.25,0,Jan-Mar,dementia,38,male,2006
209,home,41,10.25,0,Jan-Mar,dementia,93,male,2006
209,hospital,41,10.25,0,Jan-Mar,dementia,474,male,2006
209,elsehwere,41,10.25,0,Jan-Mar,dementia,28,male,2006
210,LTCF,42,10.5,1,Apr-June,dementia,36,male,2006
210,home,42,10.5,1,Apr-June,dementia,75,male,2006
210,hospital,42,10.5,1,Apr-June,dementia,429,male,2006
210,elsehwere,42,10.5,1,Apr-June,dementia,28,male,2006
211,LTCF,43,10.75,1,July-Sept,dementia,54,male,2006
211,home,43,10.75,1,July-Sept,dementia,72,male,2006
211,hospital,43,10.75,1,July-Sept,dementia,383,male,2006
211,elsehwere,43,10.75,1,July-Sept,dementia,15,male,2006
212,LTCF,44,11,1,Oct-Dec,dementia,47,male,2006
212,home,44,11,1,Oct-Dec,dementia,92,male,2006
212,hospital,44,11,1,Oct-Dec,dementia,469,male,2006
212,elsehwere,44,11,1,Oct-Dec,dementia,32,male,2006
213,LTCF,45,11.25,1,Jan-Mar,dementia,71,male,2007

213,home,45,11.25,1,Jan-Mar,dementia,101,male,2007
213,hospital,45,11.25,1,Jan-Mar,dementia,501,male,2007
213,elsehwere,45,11.25,1,Jan-Mar,dementia,55,male,2007
214,LTCF,46,11.5,1,Apr-June,dementia,54,male,2007
214,home,46,11.5,1,Apr-June,dementia,70,male,2007
214,hospital,46,11.5,1,Apr-June,dementia,458,male,2007
214,elsehwere,46,11.5,1,Apr-June,dementia,36,male,2007
215,LTCF,47,11.75,1,July-Sept,dementia,62,male,2007
215,home,47,11.75,1,July-Sept,dementia,69,male,2007
215,hospital,47,11.75,1,July-Sept,dementia,432,male,2007
215,elsehwere,47,11.75,1,July-Sept,dementia,34,male,2007
216,LTCF,48,12,1,Oct-Dec,dementia,55,male,2007
216,home,48,12,1,Oct-Dec,dementia,99,male,2007
216,hospital,48,12,1,Oct-Dec,dementia,534,male,2007
216,elsehwere,48,12,1,Oct-Dec,dementia,46,male,2007
217,LTCF,49,12.25,1,Jan-Mar,dementia,75,male,2008
217,home,49,12.25,1,Jan-Mar,dementia,103,male,2008
217,hospital,49,12.25,1,Jan-Mar,dementia,587,male,2008
217,elsehwere,49,12.25,1,Jan-Mar,dementia,56,male,2008
218,LTCF,50,12.5,1,Apr-June,dementia,70,male,2008
218,home,50,12.5,1,Apr-June,dementia,73,male,2008
218,hospital,50,12.5,1,Apr-June,dementia,512,male,2008
218,elsehwere,50,12.5,1,Apr-June,dementia,46,male,2008
219,LTCF,51,12.75,1,July-Sept,dementia,81,male,2008
219,home,51,12.75,1,July-Sept,dementia,92,male,2008
219,hospital,51,12.75,1,July-Sept,dementia,508,male,2008
219,elsehwere,51,12.75,1,July-Sept,dementia,50,male,2008
220,LTCF,52,13,1,Oct-Dec,dementia,98,male,2008
220,home,52,13,1,Oct-Dec,dementia,130,male,2008
220,hospital,52,13,1,Oct-Dec,dementia,577,male,2008
220,elsehwere,52,13,1,Oct-Dec,dementia,67,male,2008
221,LTCF,53,13.25,1,Jan-Mar,dementia,93,male,2009
221,home,53,13.25,1,Jan-Mar,dementia,130,male,2009
221,hospital,53,13.25,1,Jan-Mar,dementia,664,male,2009
221,elsehwere,53,13.25,1,Jan-Mar,dementia,58,male,2009
222,LTCF,54,13.5,1,Apr-June,dementia,100,male,2009
222,home,54,13.5,1,Apr-June,dementia,89,male,2009
222,hospital,54,13.5,1,Apr-June,dementia,557,male,2009
222,elsehwere,54,13.5,1,Apr-June,dementia,56,male,2009
223,LTCF,55,13.75,1,July-Sept,dementia,87,male,2009
223,home,55,13.75,1,July-Sept,dementia,77,male,2009
223,hospital,55,13.75,1,July-Sept,dementia,585,male,2009
223,elsehwere,55,13.75,1,July-Sept,dementia,61,male,2009
224,LTCF,56,14,1,Oct-Dec,dementia,94,male,2009
224,home,56,14,1,Oct-Dec,dementia,102,male,2009
224,hospital,56,14,1,Oct-Dec,dementia,655,male,2009
224,elsehwere,56,14,1,Oct-Dec,dementia,70,male,2009
225,LTCF,57,14.25,1,Jan-Mar,dementia,116,male,2010
225,home,57,14.25,1,Jan-Mar,dementia,112,male,2010
225,hospital,57,14.25,1,Jan-Mar,dementia,656,male,2010

225,elsehwere,57,14.25,1,Jan-Mar,dementia,83,male,2010
226,LTCF,58,14.5,1,Apr-June,dementia,115,male,2010
226,home,58,14.5,1,Apr-June,dementia,96,male,2010
226,hospital,58,14.5,1,Apr-June,dementia,675,male,2010
226,elsehwere,58,14.5,1,Apr-June,dementia,56,male,2010
227,LTCF,59,14.75,1,July-Sept,dementia,93,male,2010
227,home,59,14.75,1,July-Sept,dementia,119,male,2010
227,hospital,59,14.75,1,July-Sept,dementia,697,male,2010
227,elsehwere,59,14.75,1,July-Sept,dementia,94,male,2010
228,LTCF,60,15,1,Oct-Dec,dementia,149,male,2010
228,home,60,15,1,Oct-Dec,dementia,139,male,2010
228,hospital,60,15,1,Oct-Dec,dementia,728,male,2010
228,elsehwere,60,15,1,Oct-Dec,dementia,91,male,2010
229,LTCF,61,15.25,1,Jan-Mar,dementia,170,male,2011
229,home,61,15.25,1,Jan-Mar,dementia,143,male,2011
229,hospital,61,15.25,1,Jan-Mar,dementia,852,male,2011
229,elsehwere,61,15.25,1,Jan-Mar,dementia,97,male,2011
230,LTCF,62,15.5,1,Apr-June,dementia,166,male,2011
230,home,62,15.5,1,Apr-June,dementia,111,male,2011
230,hospital,62,15.5,1,Apr-June,dementia,804,male,2011
230,elsehwere,62,15.5,1,Apr-June,dementia,101,male,2011
231,LTCF,63,15.75,1,July-Sept,dementia,140,male,2011
231,home,63,15.75,1,July-Sept,dementia,111,male,2011
231,hospital,63,15.75,1,July-Sept,dementia,806,male,2011
231,elsehwere,63,15.75,1,July-Sept,dementia,97,male,2011
232,LTCF,64,16,1,Oct-Dec,dementia,172,male,2011
232,home,64,16,1,Oct-Dec,dementia,141,male,2011
232,hospital,64,16,1,Oct-Dec,dementia,880,male,2011
232,elsehwere,64,16,1,Oct-Dec,dementia,115,male,2011
233,LTCF,65,16.25,1,Jan-Mar,dementia,177,male,2012
233,home,65,16.25,1,Jan-Mar,dementia,174,male,2012
233,hospital,65,16.25,1,Jan-Mar,dementia,961,male,2012
233,elsehwere,65,16.25,1,Jan-Mar,dementia,121,male,2012
234,LTCF,66,16.5,1,Apr-June,dementia,209,male,2012
234,home,66,16.5,1,Apr-June,dementia,126,male,2012
234,hospital,66,16.5,1,Apr-June,dementia,868,male,2012
234,elsehwere,66,16.5,1,Apr-June,dementia,120,male,2012
235,LTCF,67,16.75,1,July-Sept,dementia,198,male,2012
235,home,67,16.75,1,July-Sept,dementia,126,male,2012
235,hospital,67,16.75,1,July-Sept,dementia,899,male,2012
235,elsehwere,67,16.75,1,July-Sept,dementia,129,male,2012
236,LTCF,68,17,1,Oct-Dec,dementia,225,male,2012
236,home,68,17,1,Oct-Dec,dementia,217,male,2012
236,hospital,68,17,1,Oct-Dec,dementia,1060,male,2012
236,elsehwere,68,17,1,Oct-Dec,dementia,131,male,2012
237,LTCF,69,17.25,1,Jan-Mar,dementia,242,male,2013
237,home,69,17.25,1,Jan-Mar,dementia,202,male,2013
237,hospital,69,17.25,1,Jan-Mar,dementia,1110,male,2013
237,elsehwere,69,17.25,1,Jan-Mar,dementia,135,male,2013
238,LTCF,70,17.5,1,Apr-June,dementia,239,male,2013

```
238,home,70,17.5,1,Apr-June,dementia,161,male,2013
238,hospital,70,17.5,1,Apr-June,dementia,1052,male,2013
238,elsehwere,70,17.5,1,Apr-June,dementia,114,male,2013
239,LTCF,71,17.75,1,July-Sept,dementia,233,male,2013
239,home,71,17.75,1,July-Sept,dementia,154,male,2013
239,hospital,71,17.75,1,July-Sept,dementia,985,male,2013
239,elsehwere,71,17.75,1,July-Sept,dementia,127,male,2013
240,LTCF,72,18,1,Oct-Dec,dementia,284,male,2013
240,home,72,18,1,Oct-Dec,dementia,205,male,2013
240,hospital,72,18,1,Oct-Dec,dementia,1148,male,2013
240,elsehwere,72,18,1,Oct-Dec,dementia,179,male,2013
241,LTCF,73,18.25,1,Jan-Mar,dementia,308,male,2014
241,home,73,18.25,1,Jan-Mar,dementia,220,male,2014
241,hospital,73,18.25,1,Jan-Mar,dementia,1214,male,2014
241,elsehwere,73,18.25,1,Jan-Mar,dementia,173,male,2014
242,LTCF,74,18.5,1,Apr-June,dementia,271,male,2014
242,home,74,18.5,1,Apr-June,dementia,167,male,2014
242,hospital,74,18.5,1,Apr-June,dementia,1029,male,2014
242,elsehwere,74,18.5,1,Apr-June,dementia,167,male,2014
243,LTCF,75,18.75,1,July-Sept,dementia,281,male,2014
243,home,75,18.75,1,July-Sept,dementia,206,male,2014
243,hospital,75,18.75,1,July-Sept,dementia,1058,male,2014
243,elsehwere,75,18.75,1,July-Sept,dementia,139,male,2014
244,LTCF,76,19,1,Oct-Dec,dementia,353,male,2014
244,home,76,19,1,Oct-Dec,dementia,211,male,2014
244,hospital,76,19,1,Oct-Dec,dementia,1285,male,2014
244,elsehwere,76,19,1,Oct-Dec,dementia,202,male,2014
245,LTCF,77,19.25,1,Jan-Mar,dementia,326,male,2015
245,home,77,19.25,1,Jan-Mar,dementia,204,male,2015
245,hospital,77,19.25,1,Jan-Mar,dementia,1282,male,2015
245,elsehwere,77,19.25,1,Jan-Mar,dementia,206,male,2015
246,LTCF,78,19.5,1,Apr-June,dementia,337,male,2015
246,home,78,19.5,1,Apr-June,dementia,185,male,2015
246,hospital,78,19.5,1,Apr-June,dementia,1174,male,2015
246,elsehwere,78,19.5,1,Apr-June,dementia,179,male,2015
247,LTCF,79,19.75,1,July-Sept,dementia,308,male,2015
247,home,79,19.75,1,July-Sept,dementia,189,male,2015
247,hospital,79,19.75,1,July-Sept,dementia,1177,male,2015
247,elsehwere,79,19.75,1,July-Sept,dementia,162,male,2015
248,LTCF,80,20,1,Oct-Dec,dementia,387,male,2015
248,home,80,20,1,Oct-Dec,dementia,244,male,2015
248,hospital,80,20,1,Oct-Dec,dementia,1344,male,2015
248,elsehwere,80,20,1,Oct-Dec,dementia,196,male,2015
249,LTCF,81,20.25,1,Jan-Mar,dementia,412,male,2016
249,home,81,20.25,1,Jan-Mar,dementia,236,male,2016
249,hospital,81,20.25,1,Jan-Mar,dementia,1374,male,2016
249,elsehwere,81,20.25,1,Jan-Mar,dementia,253,male,2016
250,LTCF,82,20.5,1,Apr-June,dementia,370,male,2016
250,home,82,20.5,1,Apr-June,dementia,197,male,2016
250,hospital,82,20.5,1,Apr-June,dementia,1227,male,2016
```

250,elsehwere,82,20.5,1,Apr-June,dementia,214,male,2016
251,LTCF,83,20.75,1,July-Sept,dementia,367,male,2016
251,home,83,20.75,1,July-Sept,dementia,202,male,2016
251,hospital,83,20.75,1,July-Sept,dementia,1269,male,2016
251,elsehwere,83,20.75,1,July-Sept,dementia,202,male,2016
252,LTCF,84,21,1,Oct-Dec,dementia,439,male,2016
252,home,84,21,1,Oct-Dec,dementia,271,male,2016
252,hospital,84,21,1,Oct-Dec,dementia,1492,male,2016
252,elsehwere,84,21,1,Oct-Dec,dementia,214,male,2016
253,LTCF,1,.25,0,Jan-Mar,dementia,59,female,1996
253,home,1,.25,0,Jan-Mar,dementia,258,female,1996
253,hospital,1,.25,0,Jan-Mar,dementia,287,female,1996
253,elsehwere,1,.25,0,Jan-Mar,dementia,24,female,1996
254,LTCF,2,.5,0,Apr-June,dementia,54,female,1996
254,home,2,.5,0,Apr-June,dementia,161,female,1996
254,hospital,2,.5,0,Apr-June,dementia,218,female,1996
254,elsehwere,2,.5,0,Apr-June,dementia,12,female,1996
255,LTCF,3,.75,0,July-Sept,dementia,43,female,1996
255,home,3,.75,0,July-Sept,dementia,155,female,1996
255,hospital,3,.75,0,July-Sept,dementia,205,female,1996
255,elsehwere,3,.75,0,July-Sept,dementia,13,female,1996
256,LTCF,4,1,0,Oct-Dec,dementia,66,female,1996
256,home,4,1,0,Oct-Dec,dementia,184,female,1996
256,hospital,4,1,0,Oct-Dec,dementia,266,female,1996
256,elsehwere,4,1,0,Oct-Dec,dementia,10,female,1996
257,LTCF,5,1.25,0,Jan-Mar,dementia,68,female,1997
257,home,5,1.25,0,Jan-Mar,dementia,249,female,1997
257,hospital,5,1.25,0,Jan-Mar,dementia,278,female,1997
257,elsehwere,5,1.25,0,Jan-Mar,dementia,17,female,1997
258,LTCF,6,1.5,0,Apr-June,dementia,38,female,1997
258,home,6,1.5,0,Apr-June,dementia,139,female,1997
258,hospital,6,1.5,0,Apr-June,dementia,261,female,1997
258,elsehwere,6,1.5,0,Apr-June,dementia,13,female,1997
259,LTCF,7,1.75,0,July-Sept,dementia,44,female,1997
259,home,7,1.75,0,July-Sept,dementia,130,female,1997
259,hospital,7,1.75,0,July-Sept,dementia,216,female,1997
259,elsehwere,7,1.75,0,July-Sept,dementia,15,female,1997
260,LTCF,8,2,0,Oct-Dec,dementia,71,female,1997
260,home,8,2,0,Oct-Dec,dementia,191,female,1997
260,hospital,8,2,0,Oct-Dec,dementia,248,female,1997
260,elsehwere,8,2,0,Oct-Dec,dementia,20,female,1997
261,LTCF,9,2.25,0,Jan-Mar,dementia,81,female,1998
261,home,9,2.25,0,Jan-Mar,dementia,254,female,1998
261,hospital,9,2.25,0,Jan-Mar,dementia,292,female,1998
261,elsehwere,9,2.25,0,Jan-Mar,dementia,21,female,1998
262,LTCF,10,2.5,0,Apr-June,dementia,55,female,1998
262,home,10,2.5,0,Apr-June,dementia,137,female,1998
262,hospital,10,2.5,0,Apr-June,dementia,220,female,1998
262,elsehwere,10,2.5,0,Apr-June,dementia,15,female,1998
263,LTCF,11,2.75,0,July-Sept,dementia,52,female,1998

263,home,11,2.75,0,July-Sept,dementia,131,female,1998
263,hospital,11,2.75,0,July-Sept,dementia,206,female,1998
263,elsehwere,11,2.75,0,July-Sept,dementia,24,female,1998
264,LTCF,12,3,0,Oct-Dec,dementia,75,female,1998
264,home,12,3,0,Oct-Dec,dementia,193,female,1998
264,hospital,12,3,0,Oct-Dec,dementia,289,female,1998
264,elsehwere,12,3,0,Oct-Dec,dementia,20,female,1998
265,LTCF,13,3.25,0,Jan-Mar,dementia,83,female,1999
265,home,13,3.25,0,Jan-Mar,dementia,244,female,1999
265,hospital,13,3.25,0,Jan-Mar,dementia,380,female,1999
265,elsehwere,13,3.25,0,Jan-Mar,dementia,44,female,1999
266,LTCF,14,3.5,0,Apr-June,dementia,74,female,1999
266,home,14,3.5,0,Apr-June,dementia,143,female,1999
266,hospital,14,3.5,0,Apr-June,dementia,314,female,1999
266,elsehwere,14,3.5,0,Apr-June,dementia,22,female,1999
267,LTCF,15,3.75,0,July-Sept,dementia,68,female,1999
267,home,15,3.75,0,July-Sept,dementia,131,female,1999
267,hospital,15,3.75,0,July-Sept,dementia,263,female,1999
267,elsehwere,15,3.75,0,July-Sept,dementia,25,female,1999
268,LTCF,16,4,0,Oct-Dec,dementia,84,female,1999
268,home,16,4,0,Oct-Dec,dementia,206,female,1999
268,hospital,16,4,0,Oct-Dec,dementia,351,female,1999
268,elsehwere,16,4,0,Oct-Dec,dementia,13,female,1999
269,LTCF,17,4.25,0,Jan-Mar,dementia,110,female,2000
269,home,17,4.25,0,Jan-Mar,dementia,262,female,2000
269,hospital,17,4.25,0,Jan-Mar,dementia,378,female,2000
269,elsehwere,17,4.25,0,Jan-Mar,dementia,39,female,2000
270,LTCF,18,4.5,0,Apr-June,dementia,94,female,2000
270,home,18,4.5,0,Apr-June,dementia,173,female,2000
270,hospital,18,4.5,0,Apr-June,dementia,330,female,2000
270,elsehwere,18,4.5,0,Apr-June,dementia,27,female,2000
271,LTCF,19,4.75,0,July-Sept,dementia,98,female,2000
271,home,19,4.75,0,July-Sept,dementia,118,female,2000
271,hospital,19,4.75,0,July-Sept,dementia,307,female,2000
271,elsehwere,19,4.75,0,July-Sept,dementia,30,female,2000
272,LTCF,20,5,0,Oct-Dec,dementia,103,female,2000
272,home,20,5,0,Oct-Dec,dementia,213,female,2000
272,hospital,20,5,0,Oct-Dec,dementia,364,female,2000
272,elsehwere,20,5,0,Oct-Dec,dementia,50,female,2000
273,LTCF,21,5.25,0,Jan-Mar,dementia,137,female,2001
273,home,21,5.25,0,Jan-Mar,dementia,236,female,2001
273,hospital,21,5.25,0,Jan-Mar,dementia,437,female,2001
273,elsehwere,21,5.25,0,Jan-Mar,dementia,46,female,2001
274,LTCF,22,5.5,0,Apr-June,dementia,111,female,2001
274,home,22,5.5,0,Apr-June,dementia,170,female,2001
274,hospital,22,5.5,0,Apr-June,dementia,343,female,2001
274,elsehwere,22,5.5,0,Apr-June,dementia,46,female,2001
275,LTCF,23,5.75,0,July-Sept,dementia,103,female,2001
275,home,23,5.75,0,July-Sept,dementia,132,female,2001
275,hospital,23,5.75,0,July-Sept,dementia,392,female,2001

275,elsehwere,23,5.75,0,July-Sept,dementia,40,female,2001
276,LTCF,24,6,0,Oct-Dec,dementia,148,female,2001
276,home,24,6,0,Oct-Dec,dementia,196,female,2001
276,hospital,24,6,0,Oct-Dec,dementia,416,female,2001
276,elsehwere,24,6,0,Oct-Dec,dementia,56,female,2001
277,LTCF,25,6.25,0,Jan-Mar,dementia,165,female,2002
277,home,25,6.25,0,Jan-Mar,dementia,209,female,2002
277,hospital,25,6.25,0,Jan-Mar,dementia,405,female,2002
277,elsehwere,25,6.25,0,Jan-Mar,dementia,58,female,2002
278,LTCF,26,6.5,0,Apr-June,dementia,121,female,2002
278,home,26,6.5,0,Apr-June,dementia,118,female,2002
278,hospital,26,6.5,0,Apr-June,dementia,390,female,2002
278,elsehwere,26,6.5,0,Apr-June,dementia,47,female,2002
279,LTCF,27,6.75,0,July-Sept,dementia,98,female,2002
279,home,27,6.75,0,July-Sept,dementia,143,female,2002
279,hospital,27,6.75,0,July-Sept,dementia,383,female,2002
279,elsehwere,27,6.75,0,July-Sept,dementia,46,female,2002
280,LTCF,28,7,0,Oct-Dec,dementia,161,female,2002
280,home,28,7,0,Oct-Dec,dementia,190,female,2002
280,hospital,28,7,0,Oct-Dec,dementia,469,female,2002
280,elsehwere,28,7,0,Oct-Dec,dementia,55,female,2002
281,LTCF,29,7.25,0,Jan-Mar,dementia,139,female,2003
281,home,29,7.25,0,Jan-Mar,dementia,210,female,2003
281,hospital,29,7.25,0,Jan-Mar,dementia,450,female,2003
281,elsehwere,29,7.25,0,Jan-Mar,dementia,62,female,2003
282,LTCF,30,7.5,0,Apr-June,dementia,145,female,2003
282,home,30,7.5,0,Apr-June,dementia,132,female,2003
282,hospital,30,7.5,0,Apr-June,dementia,401,female,2003
282,elsehwere,30,7.5,0,Apr-June,dementia,65,female,2003
283,LTCF,31,7.75,0,July-Sept,dementia,124,female,2003
283,home,31,7.75,0,July-Sept,dementia,147,female,2003
283,hospital,31,7.75,0,July-Sept,dementia,384,female,2003
283,elsehwere,31,7.75,0,July-Sept,dementia,44,female,2003
284,LTCF,32,8,0,Oct-Dec,dementia,147,female,2003
284,home,32,8,0,Oct-Dec,dementia,156,female,2003
284,hospital,32,8,0,Oct-Dec,dementia,480,female,2003
284,elsehwere,32,8,0,Oct-Dec,dementia,56,female,2003
285,LTCF,33,8.25,0,Jan-Mar,dementia,183,female,2004
285,home,33,8.25,0,Jan-Mar,dementia,223,female,2004
285,hospital,33,8.25,0,Jan-Mar,dementia,495,female,2004
285,elsehwere,33,8.25,0,Jan-Mar,dementia,79,female,2004
286,LTCF,34,8.5,0,Apr-June,dementia,144,female,2004
286,home,34,8.5,0,Apr-June,dementia,128,female,2004
286,hospital,34,8.5,0,Apr-June,dementia,487,female,2004
286,elsehwere,34,8.5,0,Apr-June,dementia,51,female,2004
287,LTCF,35,8.75,0,July-Sept,dementia,153,female,2004
287,home,35,8.75,0,July-Sept,dementia,135,female,2004
287,hospital,35,8.75,0,July-Sept,dementia,502,female,2004
287,elsehwere,35,8.75,0,July-Sept,dementia,70,female,2004
288,LTCF,36,9,0,Oct-Dec,dementia,186,female,2004

288,home,36,9,0,Oct-Dec,dementia,158,female,2004
288,hospital,36,9,0,Oct-Dec,dementia,553,female,2004
288,elsehwere,36,9,0,Oct-Dec,dementia,78,female,2004
289,LTCF,37,9.25,0,Jan-Mar,dementia,231,female,2005
289,home,37,9.25,0,Jan-Mar,dementia,198,female,2005
289,hospital,37,9.25,0,Jan-Mar,dementia,576,female,2005
289,elsehwere,37,9.25,0,Jan-Mar,dementia,72,female,2005
290,LTCF,38,9.5,0,Apr-June,dementia,188,female,2005
290,home,38,9.5,0,Apr-June,dementia,120,female,2005
290,hospital,38,9.5,0,Apr-June,dementia,528,female,2005
290,elsehwere,38,9.5,0,Apr-June,dementia,80,female,2005
291,LTCF,39,9.75,0,July-Sept,dementia,169,female,2005
291,home,39,9.75,0,July-Sept,dementia,113,female,2005
291,hospital,39,9.75,0,July-Sept,dementia,470,female,2005
291,elsehwere,39,9.75,0,July-Sept,dementia,84,female,2005
292,LTCF,40,10,0,Oct-Dec,dementia,206,female,2005
292,home,40,10,0,Oct-Dec,dementia,173,female,2005
292,hospital,40,10,0,Oct-Dec,dementia,591,female,2005
292,elsehwere,40,10,0,Oct-Dec,dementia,81,female,2005
293,LTCF,41,10.25,0,Jan-Mar,dementia,238,female,2006
293,home,41,10.25,0,Jan-Mar,dementia,228,female,2006
293,hospital,41,10.25,0,Jan-Mar,dementia,674,female,2006
293,elsehwere,41,10.25,0,Jan-Mar,dementia,94,female,2006
294,LTCF,42,10.5,1,Apr-June,dementia,226,female,2006
294,home,42,10.5,1,Apr-June,dementia,157,female,2006
294,hospital,42,10.5,1,Apr-June,dementia,564,female,2006
294,elsehwere,42,10.5,1,Apr-June,dementia,86,female,2006
295,LTCF,43,10.75,1,July-Sept,dementia,210,female,2006
295,home,43,10.75,1,July-Sept,dementia,157,female,2006
295,hospital,43,10.75,1,July-Sept,dementia,609,female,2006
295,elsehwere,43,10.75,1,July-Sept,dementia,110,female,2006
296,LTCF,44,11,1,Oct-Dec,dementia,250,female,2006
296,home,44,11,1,Oct-Dec,dementia,174,female,2006
296,hospital,44,11,1,Oct-Dec,dementia,696,female,2006
296,elsehwere,44,11,1,Oct-Dec,dementia,122,female,2006
297,LTCF,45,11.25,1,Jan-Mar,dementia,292,female,2007
297,home,45,11.25,1,Jan-Mar,dementia,242,female,2007
297,hospital,45,11.25,1,Jan-Mar,dementia,705,female,2007
297,elsehwere,45,11.25,1,Jan-Mar,dementia,114,female,2007
298,LTCF,46,11.5,1,Apr-June,dementia,252,female,2007
298,home,46,11.5,1,Apr-June,dementia,159,female,2007
298,hospital,46,11.5,1,Apr-June,dementia,667,female,2007
298,elsehwere,46,11.5,1,Apr-June,dementia,107,female,2007
299,LTCF,47,11.75,1,July-Sept,dementia,281,female,2007
299,home,47,11.75,1,July-Sept,dementia,132,female,2007
299,hospital,47,11.75,1,July-Sept,dementia,712,female,2007
299,elsehwere,47,11.75,1,July-Sept,dementia,135,female,2007
300,LTCF,48,12,1,Oct-Dec,dementia,310,female,2007
300,home,48,12,1,Oct-Dec,dementia,229,female,2007
300,hospital,48,12,1,Oct-Dec,dementia,799,female,2007

300,elsehwere,48,12,1,Oct-Dec,dementia,160,female,2007
301,LTCF,49,12.25,1,Jan-Mar,dementia,358,female,2008
301,home,49,12.25,1,Jan-Mar,dementia,249,female,2008
301,hospital,49,12.25,1,Jan-Mar,dementia,884,female,2008
301,elsehwere,49,12.25,1,Jan-Mar,dementia,156,female,2008
302,LTCF,50,12.5,1,Apr-June,dementia,310,female,2008
302,home,50,12.5,1,Apr-June,dementia,204,female,2008
302,hospital,50,12.5,1,Apr-June,dementia,746,female,2008
302,elsehwere,50,12.5,1,Apr-June,dementia,148,female,2008
303,LTCF,51,12.75,1,July-Sept,dementia,343,female,2008
303,home,51,12.75,1,July-Sept,dementia,198,female,2008
303,hospital,51,12.75,1,July-Sept,dementia,770,female,2008
303,elsehwere,51,12.75,1,July-Sept,dementia,150,female,2008
304,LTCF,52,13,1,Oct-Dec,dementia,380,female,2008
304,home,52,13,1,Oct-Dec,dementia,253,female,2008
304,hospital,52,13,1,Oct-Dec,dementia,855,female,2008
304,elsehwere,52,13,1,Oct-Dec,dementia,180,female,2008
305,LTCF,53,13.25,1,Jan-Mar,dementia,413,female,2009
305,home,53,13.25,1,Jan-Mar,dementia,267,female,2009
305,hospital,53,13.25,1,Jan-Mar,dementia,858,female,2009
305,elsehwere,53,13.25,1,Jan-Mar,dementia,199,female,2009
306,LTCF,54,13.5,1,Apr-June,dementia,383,female,2009
306,home,54,13.5,1,Apr-June,dementia,211,female,2009
306,hospital,54,13.5,1,Apr-June,dementia,763,female,2009
306,elsehwere,54,13.5,1,Apr-June,dementia,161,female,2009
307,LTCF,55,13.75,1,July-Sept,dementia,416,female,2009
307,home,55,13.75,1,July-Sept,dementia,166,female,2009
307,hospital,55,13.75,1,July-Sept,dementia,837,female,2009
307,elsehwere,55,13.75,1,July-Sept,dementia,195,female,2009
308,LTCF,56,14,1,Oct-Dec,dementia,452,female,2009
308,home,56,14,1,Oct-Dec,dementia,269,female,2009
308,hospital,56,14,1,Oct-Dec,dementia,952,female,2009
308,elsehwere,56,14,1,Oct-Dec,dementia,194,female,2009
309,LTCF,57,14.25,1,Jan-Mar,dementia,488,female,2010
309,home,57,14.25,1,Jan-Mar,dementia,269,female,2010
309,hospital,57,14.25,1,Jan-Mar,dementia,877,female,2010
309,elsehwere,57,14.25,1,Jan-Mar,dementia,257,female,2010
310,LTCF,58,14.5,1,Apr-June,dementia,480,female,2010
310,home,58,14.5,1,Apr-June,dementia,205,female,2010
310,hospital,58,14.5,1,Apr-June,dementia,935,female,2010
310,elsehwere,58,14.5,1,Apr-June,dementia,237,female,2010
311,LTCF,59,14.75,1,July-Sept,dementia,520,female,2010
311,home,59,14.75,1,July-Sept,dementia,215,female,2010
311,hospital,59,14.75,1,July-Sept,dementia,974,female,2010
311,elsehwere,59,14.75,1,July-Sept,dementia,304,female,2010
312,LTCF,60,15,1,Oct-Dec,dementia,592,female,2010
312,home,60,15,1,Oct-Dec,dementia,294,female,2010
312,hospital,60,15,1,Oct-Dec,dementia,1054,female,2010
312,elsehwere,60,15,1,Oct-Dec,dementia,291,female,2010
313,LTCF,61,15.25,1,Jan-Mar,dementia,668,female,2011

313,home,61,15.25,1,Jan-Mar,dementia,362,female,2011
313,hospital,61,15.25,1,Jan-Mar,dementia,1177,female,2011
313,elsehwere,61,15.25,1,Jan-Mar,dementia,353,female,2011
314,LTCF,62,15.5,1,Apr-June,dementia,635,female,2011
314,home,62,15.5,1,Apr-June,dementia,272,female,2011
314,hospital,62,15.5,1,Apr-June,dementia,1198,female,2011
314,elsehwere,62,15.5,1,Apr-June,dementia,298,female,2011
315,LTCF,63,15.75,1,July-Sept,dementia,685,female,2011
315,home,63,15.75,1,July-Sept,dementia,238,female,2011
315,hospital,63,15.75,1,July-Sept,dementia,1081,female,2011
315,elsehwere,63,15.75,1,July-Sept,dementia,316,female,2011
316,LTCF,64,16,1,Oct-Dec,dementia,743,female,2011
316,home,64,16,1,Oct-Dec,dementia,302,female,2011
316,hospital,64,16,1,Oct-Dec,dementia,1207,female,2011
316,elsehwere,64,16,1,Oct-Dec,dementia,367,female,2011
317,LTCF,65,16.25,1,Jan-Mar,dementia,876,female,2012
317,home,65,16.25,1,Jan-Mar,dementia,413,female,2012
317,hospital,65,16.25,1,Jan-Mar,dementia,1387,female,2012
317,elsehwere,65,16.25,1,Jan-Mar,dementia,433,female,2012
318,LTCF,66,16.5,1,Apr-June,dementia,716,female,2012
318,home,66,16.5,1,Apr-June,dementia,295,female,2012
318,hospital,66,16.5,1,Apr-June,dementia,1202,female,2012
318,elsehwere,66,16.5,1,Apr-June,dementia,371,female,2012
319,LTCF,67,16.75,1,July-Sept,dementia,819,female,2012
319,home,67,16.75,1,July-Sept,dementia,274,female,2012
319,hospital,67,16.75,1,July-Sept,dementia,1262,female,2012
319,elsehwere,67,16.75,1,July-Sept,dementia,408,female,2012
320,LTCF,68,17,1,Oct-Dec,dementia,996,female,2012
320,home,68,17,1,Oct-Dec,dementia,443,female,2012
320,hospital,68,17,1,Oct-Dec,dementia,1582,female,2012
320,elsehwere,68,17,1,Oct-Dec,dementia,496,female,2012
321,LTCF,69,17.25,1,Jan-Mar,dementia,991,female,2013
321,home,69,17.25,1,Jan-Mar,dementia,480,female,2013
321,hospital,69,17.25,1,Jan-Mar,dementia,1619,female,2013
321,elsehwere,69,17.25,1,Jan-Mar,dementia,467,female,2013
322,LTCF,70,17.5,1,Apr-June,dementia,975,female,2013
322,home,70,17.5,1,Apr-June,dementia,346,female,2013
322,hospital,70,17.5,1,Apr-June,dementia,1442,female,2013
322,elsehwere,70,17.5,1,Apr-June,dementia,438,female,2013
323,LTCF,71,17.75,1,July-Sept,dementia,979,female,2013
323,home,71,17.75,1,July-Sept,dementia,343,female,2013
323,hospital,71,17.75,1,July-Sept,dementia,1483,female,2013
323,elsehwere,71,17.75,1,July-Sept,dementia,427,female,2013
324,LTCF,72,18,1,Oct-Dec,dementia,1249,female,2013
324,home,72,18,1,Oct-Dec,dementia,462,female,2013
324,hospital,72,18,1,Oct-Dec,dementia,1709,female,2013
324,elsehwere,72,18,1,Oct-Dec,dementia,514,female,2013
325,LTCF,73,18.25,1,Jan-Mar,dementia,1182,female,2014
325,home,73,18.25,1,Jan-Mar,dementia,458,female,2014
325,hospital,73,18.25,1,Jan-Mar,dementia,1782,female,2014

325,elsehwere,73,18.25,1,Jan-Mar,dementia,536,female,2014
326,LTCF,74,18.5,1,Apr-June,dementia,1065,female,2014
326,home,74,18.5,1,Apr-June,dementia,356,female,2014
326,hospital,74,18.5,1,Apr-June,dementia,1587,female,2014
326,elsehwere,74,18.5,1,Apr-June,dementia,484,female,2014
327,LTCF,75,18.75,1,July-Sept,dementia,1126,female,2014
327,home,75,18.75,1,July-Sept,dementia,377,female,2014
327,hospital,75,18.75,1,July-Sept,dementia,1601,female,2014
327,elsehwere,75,18.75,1,July-Sept,dementia,510,female,2014
328,LTCF,76,19,1,Oct-Dec,dementia,1355,female,2014
328,home,76,19,1,Oct-Dec,dementia,462,female,2014
328,hospital,76,19,1,Oct-Dec,dementia,1820,female,2014
328,elsehwere,76,19,1,Oct-Dec,dementia,588,female,2014
329,LTCF,77,19.25,1,Jan-Mar,dementia,1366,female,2015
329,home,77,19.25,1,Jan-Mar,dementia,527,female,2015
329,hospital,77,19.25,1,Jan-Mar,dementia,1874,female,2015
329,elsehwere,77,19.25,1,Jan-Mar,dementia,605,female,2015
330,LTCF,78,19.5,1,Apr-June,dementia,1195,female,2015
330,home,78,19.5,1,Apr-June,dementia,360,female,2015
330,hospital,78,19.5,1,Apr-June,dementia,1673,female,2015
330,elsehwere,78,19.5,1,Apr-June,dementia,600,female,2015
331,LTCF,79,19.75,1,July-Sept,dementia,1243,female,2015
331,home,79,19.75,1,July-Sept,dementia,385,female,2015
331,hospital,79,19.75,1,July-Sept,dementia,1716,female,2015
331,elsehwere,79,19.75,1,July-Sept,dementia,577,female,2015
332,LTCF,80,20,1,Oct-Dec,dementia,1524,female,2015
332,home,80,20,1,Oct-Dec,dementia,498,female,2015
332,hospital,80,20,1,Oct-Dec,dementia,1959,female,2015
332,elsehwere,80,20,1,Oct-Dec,dementia,635,female,2015
333,LTCF,81,20.25,1,Jan-Mar,dementia,1602,female,2016
333,home,81,20.25,1,Jan-Mar,dementia,527,female,2016
333,hospital,81,20.25,1,Jan-Mar,dementia,1919,female,2016
333,elsehwere,81,20.25,1,Jan-Mar,dementia,633,female,2016
334,LTCF,82,20.5,1,Apr-June,dementia,1417,female,2016
334,home,82,20.5,1,Apr-June,dementia,424,female,2016
334,hospital,82,20.5,1,Apr-June,dementia,1773,female,2016
334,elsehwere,82,20.5,1,Apr-June,dementia,616,female,2016
335,LTCF,83,20.75,1,July-Sept,dementia,1404,female,2016
335,home,83,20.75,1,July-Sept,dementia,455,female,2016
335,hospital,83,20.75,1,July-Sept,dementia,1862,female,2016
335,elsehwere,83,20.75,1,July-Sept,dementia,571,female,2016
336,LTCF,84,21,1,Oct-Dec,dementia,1825,female,2016
336,home,84,21,1,Oct-Dec,dementia,562,female,2016
336,hospital,84,21,1,Oct-Dec,dementia,2155,female,2016
336,elsehwere,84,21,1,Oct-Dec,dementia,703,female,2016
