# Supplementary material for: The Revised Medical Care Act is associated with a decrease in hospital death for the total Japanese older adult population regardless of dementia status: An interrupted time series analysis
Source: PLoS One. 2022 Mar 3;17(3):e0264624. doi: 10.1371/journal.pone.0264624 (PMC8893620; doi:10.1371/journal.pone.0264624)
Supplement: S1 Appendix — (DOCX) [file pone.0264624.s002.docx]

**S2 Appendix 1**

**Jandoc methodological and reporting recommendations for interrupted time series studies**

**Title and abstract**

1. Indicate the study design (interrupted time series) in the title or abstract.

Title: Japan’s national social and health care reform for older adults is associated with changes in location of death: an interrupted time series analysis.

**Introduction**

1. *Background/rationale*

Provide background regarding the intervention and setting under investigation to support the study

rationale and methods.

In 2006, the Japanese government implemented a major reform of the long-term care system by the enactment of the Revised Medical Care Act, to safeguard financial sustainability and improve care for its aging society. Under the new act, end-of-life care was established in the community-based care system, to enable older adults live at their own home at the end-of-life stage with dignity, adequate in-home health and nursing care. Also, a new medical insurance system for the latter-stage older adults was instituted, which included additional benefits to pay for end-of-life care in home and nursing homes.

1. *Objectives*
2. State specific objectives and any pre-specified hypotheses.

This study aimed to evaluate the immediate and continued effects of the Japanese 2006 Revised Medical Care Act on location of death for the total population and for persons with dementia using interrupted time series analyses.

1. Distinguish between primary and secondary objectives

N/A

**Methods**

1. *Intervention*

Define the intervention time point(s) used in the analysis

In an ITS outcome data is taken from regularly spaced intervals and is divided by an interruption point, in this case the introduction of the Revised Medical Care Act April 2006.

1. *Participants*
2. List eligibility criteria and methods of selection

All older Japanese adults 65 year and older

1. Define subgroups

ICD10, tenth revision, codes for dementia; Dementia in Alzheimer’s’ disease: F00.0, F00.1, F00.2, F00.9; Vascular dementia: F01.0, F01.1, F01.2, F01.3, F01.8, F01.9; Dementia in other diseases classified elsewhere F02.0, F02.1, F02.2, F02.3, F02.4, F02.8; Unspecified dementia F03; Alzheimer disease G30.0, G30.1, G30.8, G30.9

(c) Consider including a comparison group not exposed to the intervention as a secondary group of Participants

No comparison group available

1. *Data sources and measurement*
2. List data source(s)

This population-based study used death certificate data from the entire Japanese population 65 year and older, collected by vital statistics Japan. The data included monthly repeated cross-sectional, national aggregated data on location of death.

1. Comment on data completeness, validity, and changes in data coverage over time

The data was anonymized and publicly available, repeated cross-sectional, national aggregated data on location of death (hospital, nursing home, home, elsewhere). The data covered the entire Japanese population over 65 years and older.

1. *Variables*
2. Define all variables

- Outcome variable(s)

The primary outcome variable was location of death based on the death certificate categorized by hospital (reference category), nursing home, home, and elsewhere.

-Descriptive and stratifying variable(s)

Explanatory variables included a *Time* variable in cumulative quarters, a *Reform* dummy variable coded as 0 pre-reform period and 1 as post-reform period, calendar quarters as a categorical variable to control for seasonality and sex as a covariate.

1. Comment on change in variable coding over time

N/A

1. Consider including details of variable coding in supplemental material, for example, appendix or research Web site

N/A

1. *Statistical methods*
2. Report all statistical methods.

- Study time intervals, for example, monthly, quarterly:

quarterly

- Regression model, for example, ARIMA, linear, segmented:

Multinomial logistic regression model

- Number of data points pre-intervention, post-intervention, and between intervention

Pre-intervention points: 41-year quarters from January 1996 until March 2006

Post-intervention points: 43-year quarters from April 2006 until December 2016

No between intervention points

1. Indicate how autocorrelation, non-stationarity, and seasonality were tested and handled:

Calendar quarters were included as a categorical variable in the model to account for seasonality

1. Consider a lag period if intervention effects are gradual or delayed:

The time point of the reforms was set immediately after the reforms (enacted April 2006). No time lag was expected because financial incentives of the reforms took effect immediately.

1. Define and distinguish between primary and secondary or sensitivity analyses:

We conducted sensitivity analysis on adjustment for seasonality by including calendar months, yearly quarters, and Fourier terms (sine cosine functions).

1. Consider use of comparison outcome(s) and/or population(s) not exposed to the intervention(s) as secondary analyses:

No comparison group available

1. Report statistical software used for analysis:

Stata version 16 was used for all analyses.

**Results**

1. *Participants*
2. Report the number of individuals and/or observations in each group analyzed

A total of 19,307,104 older adults died in the study period (January 1, 1996 - December 31, 2016). Of these 8,035,104 occurred before the implementation of the Revised Medical Care Act. From the total population, 216,442 (1·1%) persons with dementia died in the study period, of which 44,430 died before reform.

1. Consider use of a flow diagram.

N/A

1. Describe characteristics and indicate missing data:

We have no info on missing data, data consisted of total Japanese population >65 years

1. *Outcome data*
2. Report the number of outcomes examined over the study period.

19,307,104 locations of death over the total study period

1. Report the average, minimum, and maximum number of outcomes across time intervals.

Average, minimum, and maximum ranges per quarter were 14365, 5 to 126,625 for the total population and 5 to 2155 for persons with dementia.

1. Report on data variability.

NA

1. Comment on outliers and ceiling or floor effects where relevant.

N/A

1. *Main results*
2. Present results using a graphical display with intervention time point(s) clearly defined.

See figure one

1. Consider including forecasted results graphically.

We did not provide counterfactual in the figure

1. Report absolute and/or relative change(s) and their significance, for example, clinical or policy and statistical.

Total population

Pre-reform trends indicated a significant increase in the proportion of death in nursing homes (cumulative quarters, aRRR 1·02, 95% CI 1·02-1·02) and elsewhere (aRRR 1·01, 95% CI 1·00-1·01) while home death decreased (aRRR 0·94, 95% CI 0·94-0·94) compared to hospital death (figure 1 and Table 2). After the reform, we found a negative step change in the proportion of death in nursing homes (aRRR 0·37, 95% CI 0·37-0·38), at home (aRRR 0·49, 95% CI 0·48-0·49), and elsewhere (aRRR 0·53, 95% CI 0·52-0·54). Post-reform trend compared to pre-reform secular trend we found evidence of a gradual acceleration (slope change) for death in nursing homes (aRRR 1·10, 95% CI 1·10-1·10), home (aRRR 1·08, 95% CI 1·08-1·08), and elsewhere (aRRR 1·07, 95% CI 1·07-1·07). Males were more likely than females to die in a hospital compared to all other locations of death.

Persons with dementia

Pre-reform trends indicate a relative increase in the proportions of nursing home death (aRRR 1·03, 95% CI 1·03-1·05) and dying elsewhere (aRRR 1·07, 95% CI 1·05-1·09), while there was a decrease in home death (aRRR 0·89, 95% CI 0·88-0·89) (figure 1 and table 2). With the reform implementation, there was a negative step change in the proportion of death in nursing homes (aRRR 0·66, 95% CI 0·69-0·73) and home (aRRR 0·33, 95% CI 0·30-0·37), while there was a positive step change in death elsewhere (aRRR 1·22, 95% CI 1·05-1·40). Post-reform compared to pre-reform secular trend we found a gradual acceleration in nursing home death (aRRR 1·04, 95% CI 1·03-1·05) and home death (aRRR 1·11, 95% CI 1·10-1·12). Males were more likely than females to die in a hospital compared to all other locations of death.

1. *Other aalyses*

Report additional results (secondary and sensitivity analyses) in the article, appendix, or research Web site.

N/A

**Discussion**

1. *Key reslts*

Summarize key results with reference to study objectives.

In this nationally representative study of the total Japanese population 65 years and over, we found that the 2006 Revised Medical Care Act was associated with an increased trend change in proportions of death in nursing home, home and elsewhere for the total population. For persons with dementia, only proportions of death in nursing home and home had an increased trend change. The proportion of the total population and for persons with dementia dying in a nursing home nearly doubled since the reform, which was mostly attributed to the decrease in hospital deaths. This is a positive development considering hospitals are not favorable to spend the end of life. Nevertheless, proportions of hospital death remain notably higher than death in nursing home, home, and elsewhere for the total population and for persons with dementia. The decreasing trend of proportion of home deaths has stabilized since the implementation of the reform for the total population and for persons with dementia, making up just over one tenth of deaths for both populations. Death elsewhere remains the lowest proportion of location of death since the reform for the total population, while for persons with dementia it slightly exceeded home death.

1. *Context*

(a) Provide context related to possible confounding.

- Discuss relevant co-interventions that occurred during the study period

We recognize that the post-reform period (2006-2016) included other health reforms that may have influences changes in location of death. However, we carefully examined the post-reform year trends and did not objectify clear step or slope changes that might indicate substantial effects of other reforms. Moreover, the Revised Medical Care Act is the most impactful reform of Japanese long-term care.

- Comment on the stability of participant characteristics over time

Limitations include death certificates underestimating the number of deaths from dementia

- Comment on the stability of outcome coding over time

N/A

1. Discuss results of comparison analyses or provide a rationale if no comparison group was considered

N/A

1. *Limitatins*
2. Discuss limitations of the study.

There was no data available from death certificates if persons transferred location short before their death. Also, no sociodemographic and health related factors were included in the analysis as these were not available.

1. Comment on data variability and appropriateness of the number of data points

Strengths of this study include being the first study to evaluate effect of the Revised Medical Care Act on location of death for the total population and persons with dementia using longitudinal population-based death registry data covering the entire Japanese population.

1. Comment on ceiling or floor effects and outliers where relevant.

N/A

1. *Interpretation*

Provide overall interpretation of results considering objectives, limitations, results from similar studies, and other relevant evidence.

Results of this study provide implications for policies where focus on end-of-life care is increasingly integrated. In the years preceding 2006, health for older adults mainly focussed on medical care aspects. In contrast, the Revised Medical Care Act was characterised by integrated measures across health and social care for older adults at community level. This approach seems one of the key considerations to improve aging in place because it was successful in turning the peak of hospital death into a downward trend. However, building sufficient capacity remains an important challenge for a society that is aging rapidly. For this reason, the Revised Medical Care Act might have been suboptimal in improving aging in place on the long term, as hospital death was still very common. Availability of social care was insufficient, while it is crucial to extend the time in the community until death for older adults. As care needs of older adults increase over time, health professionals, family members or older adults might choose hospitalization in the end of life when home care, advocacy services and family support is not well integrated at community level. Another important issue was the lack of availability in nursing home placement. Moreover, once admitted to a nursing home, due to the absence of well-established end-of-life strategies such as advance care planning, older adults were likely to experience hospital transfers at the end-of-life.

**Other information**

1. *Funding*

List funding source(s) and role of funders

This study is funded by the Netherlands Organization for Health Research and Development (NWO-ZonMw Veni, 091.619.060).

1. *Referenes*

Reference methodological articles that support statistical methods used

Bernal JL, Cummins S, Gasparrini A. Interrupted time series regression for the evaluation of public health interventions: a tutorial. *Int J Epidemiol* 2017; **46**(1): 348-55.
